# Numerical and *in-vitro* experimental assessment of the performance of a novel designed expanded-polytetrafluoroethylene stentless bi-leaflet valve for aortic valve replacement

**DOI:** 10.1371/journal.pone.0210780

**Published:** 2019-01-30

**Authors:** Guangyu Zhu, Munirah Binte Ismail, Masakazu Nakao, Qi Yuan, Joon Hock Yeo

**Affiliations:** 1 School of Energy and Power Engineering, Xi’an Jiaotong University, Xian, Shaanxi, China; 2 School of Mechanical and Aerospace Engineering, Nanyang Technological University, Singapore, Singapore; 3 Cardiothoracic Surgery, KK Women’s and Children’s Hospital, Singapore, Singapore; Universita degli Studi di Bologna, ITALY

## Abstract

The expanded polytetrafluoroethylene (ePTFE) heart valve can serve as a viable option for prosthetic aortic valve. In this study, an ePTFE bi-leaflet valve design for aortic valve replacement (AVR) is presented, and the performance of the proposed valve was assessed numerically and experimentally. The valve was designed using CAE software. The dynamic behavior of the newly designed bi-leaflet valve under time-varying physiological pressure loading was first investigated by using commercial finite element code. Then, in-vitro tests were performed to validate the simulation and to assess the hemodynamic performance of the proposed design. A tri-leaflet ePTFE valve was tested *in-vitro* under the same conditions as a reference. The maximum leaflet coaptation area of the bi-leaflet valve during diastole was 216.3 mm^2^. When fully closed, no leakage gap was observed and the free edges of the molded valve formed S-shaped lines. The maximum Von Mises stress during a full cardiac cycle was 4.20 MPa. The dynamic performance of the bi-leaflet valve was validated by the *in-vitro* test under physiological aortic pressure pulse. The effective orifice area (EOA), mean pressure gradient, regurgitant volume, leakage volume and energy loss of the proposed valve were 3.14 cm^2^, 8.74 mmHg, 5.93 ml/beat, 1.55 ml/beat and 98.99 mJ, respectively. This study reports a novel bi-leaflet valve design for AVR. The performance of the proposed valve was numerically and experimentally assessed. Compared with the reference valve, the proposed design exhibited better structural and hemodynamic performances, which improved valve competency. Moreover, the performance of the bi-leaflet design is comparable to commercialized valves available on the market. The results of the present study provide a viable option for the future clinical applications.

## Introduction

Congenital birth defects such as aortic incompetence may lead to aortic failure. Nowadays, there have been a number of publications indicated the growing enthusiasm in the aortic valve repair techniques in children [1, 2]. Because of the encouraging mid- and long-term results in treating aortic stenosis or aortic regurgitation, percutaneous or surgical aortic valve repair is generally recommended as the primary management strategy in pediatric patients with aortic valve diseases [1, 3, 4]. Despite the fact that aortic valve repair has developed rapidly in pediatric patients, aortic valve replacement (AVR) may still be required in some cases, such as significant valve destruction and those after repair failure [4].

The search for an ideal aortic valve substitute has been ongoing for more than fifty years [5], and multiple mature surgical options for AVR are available for patients, including Ross procedure (pulmonary autograft), aortic homograft, mechanical prosthesis, and bio-prosthetic valves. However, the selection of a proper prosthesis for the pediatric patient can be challenging and controversial [1, 6], and each choice has its advantages and limitations.

The Ross procedure is a widely accepted surgical option for treating aortic valve failure [7–11]. In the Ross procedure, the aortic valve of the patient is removed, and the pulmonary valve (autograft) of the patient is transplanted to the aortic site. As the valve substitute is alive after implantation, it can grow with the patient. Additionally, long-term anticoagulation is not required after this procedure. However, the Ross procedure is limited by the complex surgical techniques and extensive time required, and furthermore, it is not suitable for certain patients (patients with a diseased pulmonary valve, large discrepancies between pulmonary and aortic valve sizes or a connective tissue disorder).

The aortic homograft (allograft) comes from the human donor and is thought to be a suitable substitute for patients who are too small for mechanical or bio-prosthetic valves [12]. In addition, the aortic homograft offers several advantages, including good hemodynamics, low thrombogenicity, and no anticoagulation after implantation. Nevertheless, the adoption of aortic homograft is constrained by its suboptimal durability and limited availability [13].

Bioprosthetic valves, otherwise known as tissue valves, can be derived from various sources, including porcine (pig), bovine (cow) and homografts or allografts. The main advantage of such valves is that patients do not require life-long anticoagulation. However, the use of bioprosthetic valves in patients has significant disadvantages. Tissue valves are less durable than their mechanical counterparts. The lifespan of such valves is between 8 to 20 years, after which replacement is required.

Despite the above-mentioned advantages and limitations of the available valve substitutes, the small conduit size of pediatric patients may bring along additional challenges on the valve design [14].

The good clinical outcomes of using expanded polytetrafluoroethylene (ePTFE) bi-leaflet valve substitutes for pulmonary valve replacement (PVR) in right ventricular outflow tract reconstruction (RVOT) for pediatric patients, however, provided a useful reference to overcome the challenges on the aortic site.

The ePTFE valved conduit for PVR has shown particular promise as the preeminent valve in pediatric patients. For the pediatric patients with small pulmonary artery size, ePTFE has been reportedly used for PVR not only with tricuspid configuration but also with monocuspid and bi-leaflet configurations [15]. Despite the native pulmonary valve also composed with three leaflets, positive results of the using of bi-leaflet prostheses in PVR has been widely reported [15–24]. Miyazaki et al. reported excellent outcomes of the ePTFE valves (monocuspid, bi-leaflet and tri-leaflet) for right ventricular outflow tract reconstruction (RVOT) in a multicenter study in Japan [24], of which the mean follow-up was 3.6 years (1.1 months to 10 years) and the free of reoperation rate at 10 years was above 92%. In another clinical trial, Miyazaki et al. implanted ePTFE valves (monocuspid, bi-leaflet and tri-leaflet) for RVOT in 157 patients (aged 16 days to 45.4 years, median 2 years) [14], and no mortality, morbidity or reoperation were reported during the follow-up period (5.6 to 63.7 months, mean 20.8 months). Moreover, the good biocompatibility of ePTFE artificial heart valves has been verified by several clinical studies [14, 25, 26]. In recent literature, the ePTFE membrane was also selected as the material for aortic valve extension [27].

However, the performances of the bi-leaflet ePTFE valve prostheses in the aortic site are yet to be studied. In this study, numerical simulation and *in-vitro* experiments were conducted to investigate the dynamic and hemodynamic performances of a novel designed bi-leaflet ePTFE valve prostheses under aortic loading. A fully sutured tri-leaflet valve [28, 29] was tested under the same conditions as a reference.

## Materials and methods

### Valve design

The native aortic valve has a complex geometry and structure, and thus, it is difficult to completely mimic the native valve in a prosthetic heart valve(PHV) design. Important design parameters for PHVs include effective orifice area (EOA), jet velocity, pressure gradient, regurgitation and thrombogenic potential, leaflet coaptation height and geometries of the leaflets [30].

At the beginning of this study, the range of several parameters, including the diameter (25 mm) and valve height (25-30 mm), were pre-defined by the surgeon in the team. Besides the quantitative parameters, an S-shaped free edge at the closed configuration that implies a surplus coaptation was expected [29]. The extra coaptation would play an important role to maintain the functionality of the valve when the aorta become bigger due to growth. In addition, the following design principles were obeyed in the design strategy [31]:

- Easy and steady valve preparation;
- Consistent preparation procedure;
- Minimum trans-valvular pressure drop;
- Minimum regurgitation;
- Easy to implant;
- Available in a wide range of sizes;

To achieve the goal of the design, the leaflet’s commissure edge was incorporated with the aortic root. The length of the leaflet’s free edge was carefully selected, which is important to guarantee the full coaptation and to prevent unwanted leaflet twisting. The initial position of the leaflets was set to a fully open configuration.

**Fig 1:**
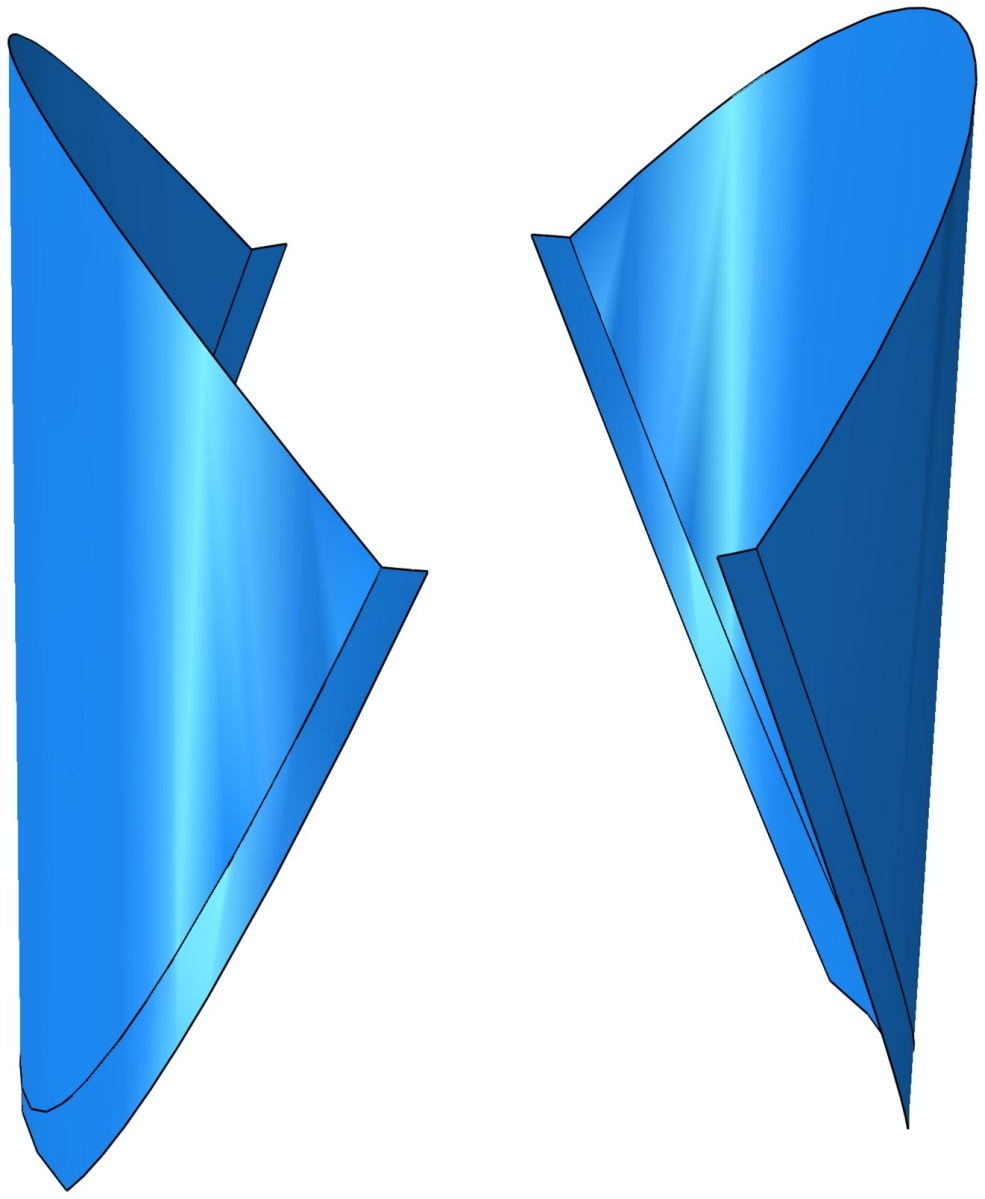
The geometry of the bi-leaflet valve.

A series of different leaflet designs based on the parameters and principles mentioned above were created in CAE software (Solidworks, Dassault Systems S.A., Paris, France). The leaflet designs were then converted to fully nonlinear finite element code ABAQUS (ABAQUS, Inc., Pawtucket, RI) to verify their function. The criterion of a successful design include: 1)the valve could properly open and close under static pressure loading applied on the leaflet surface; 2) An S-shape free edge can be observed in after the valve closed. Based on the above-listed parameters and requirements, the parametric design method was utilized in this study. Totally 5 models were generated by the engineer in our team. The one presented in Fig 1 is the only design that fully meets the above-mention requirements.

### FEM simulation

The dynamic behaviors of the molded bi-leaflet valve, including dynamic deformation, leaflet coaptation area, and stress distribution were investigated dynamically by using ABAQUS/Explicit modulus.

The ePTFE membranes were assumed to be isotropic and homogenous. An elastic modulus of 34 MPa and a density of 1100 kg/m^3^ were assigned to the ePTFE leaflet. The aortic root was modeled as a flexible hollow cylinder. The elastic modulus and density of the aortic root were set to 2 MPa and 2000 kg/m^3^, respectively [29]. Poisson’s ratio was set to 0.45 for all materials to account for the incompressible behavior of the membrane and tissues. The valve and aortic root were meshed using 4-node, doubly curved quadrilateral shell elements with reduced integration. A uniform thickness of 0.1 mm was assigned to the valve, while the thickness of the aortic root was defined to 0.4 mm.

**Fig 2:**
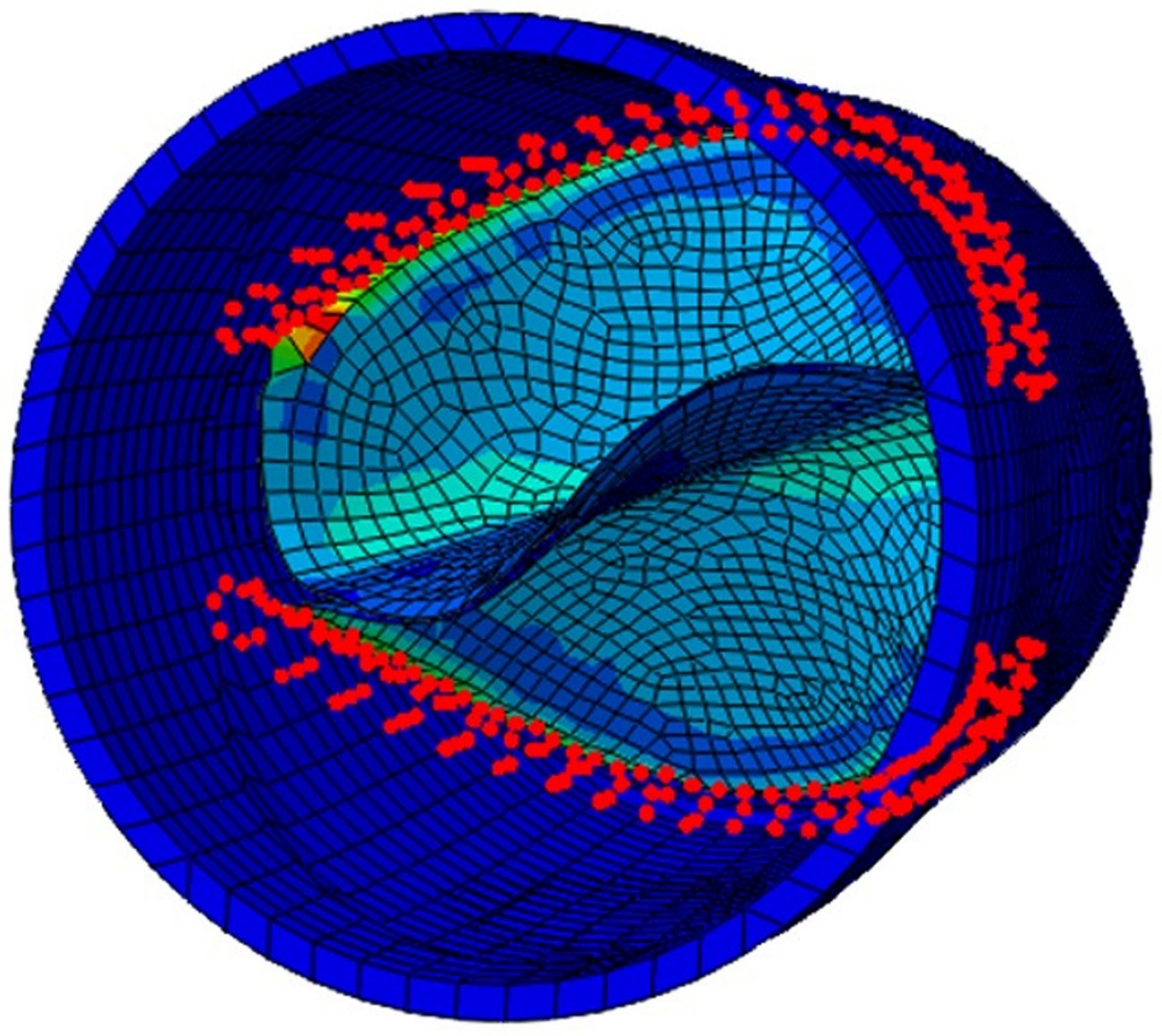
FEM model of bi-leaflet valves.

The model was assumed to be stress-free in the fully open configuration. The radial displacement of the aortic root ends was constrained. Commissures of the leaflets were connected to the aortic root by using the tie boundary condition to simulate suturing in clinical applications. Contacts between the leaflets, and between the leaflets and conduit were considered. Fig 2 shows the FEM model.

**Fig 3:**
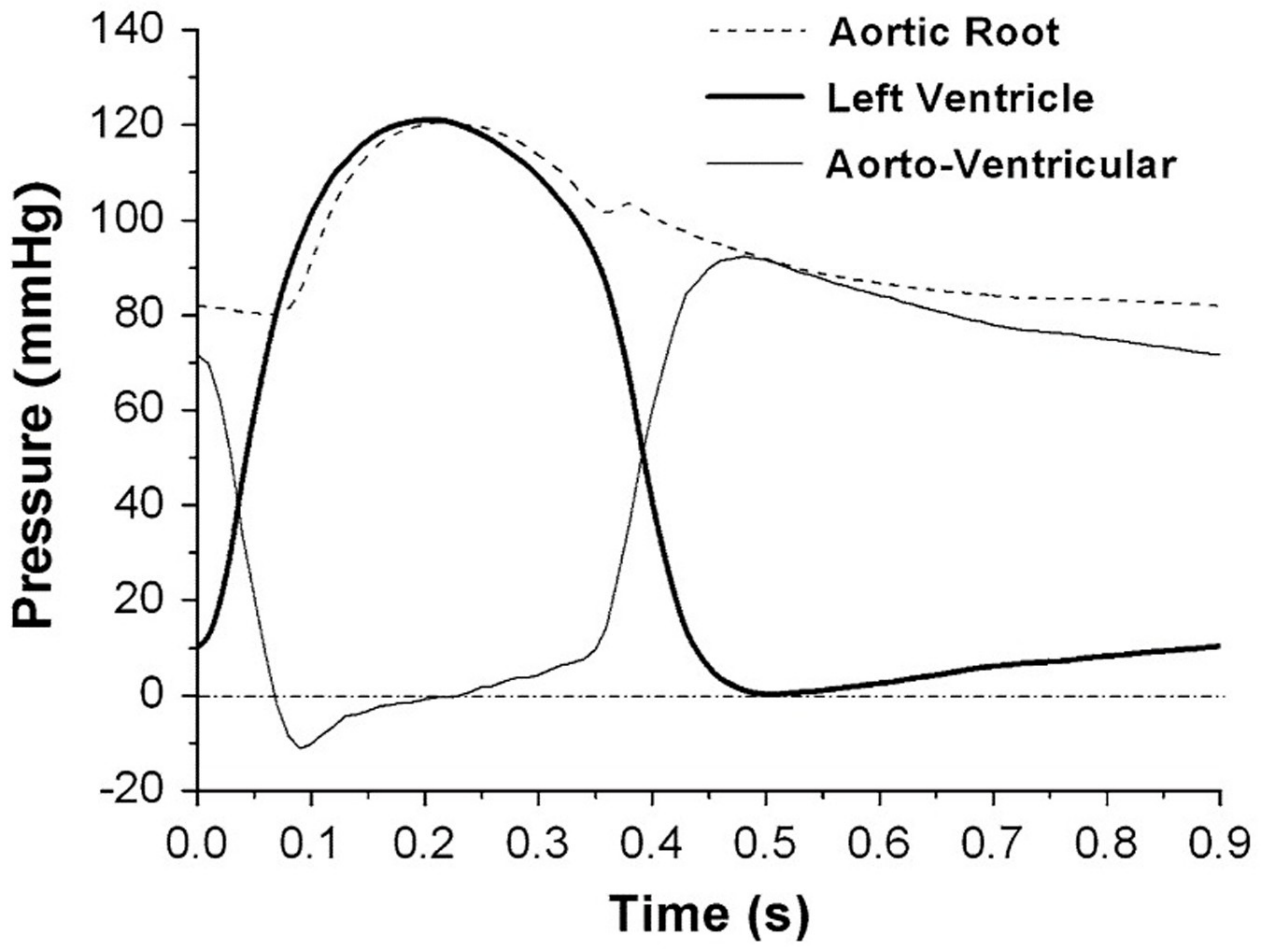
Time-varying pressure loading applied in the FEM simulation over a full cardiac cycle.

To obtain a converged solution, an aorto-ventricular pressure gradient at diastolic (71.6 mmHg) were gradually applied to the valve leaflets in 0.1s. At the end of this step, the leaflets were at a closed, diastolic, stressed configuration. Then time-varying and spatially uniform physiological aorto-ventricular pressure gradient loadings over a full cardiac cycle of 0.83 s were applied on the valve leaflets (Fig 3). In the contact model, the friction coefficient of 0.5 was set.

### *In-vitro* experiment

To assess the hemodynamic performance of the proposed design and to validate the FEM simulation, *in-vitro* experiments were carried out. According to FDA regulations, a full range of pre-clinical *in-vitro* test composed of 12 parts, which include bio-compatibility of the material, durability testing, hemodynamic performance, structural performance and fatigue assessments, etc. [32], is required. However, as the current study represents only the initial stage of valve design, only hemodynamic performance and dynamic performance were investigated in the *in-vitro* experiment.

#### Preparation of physical models

**Fig 4:**
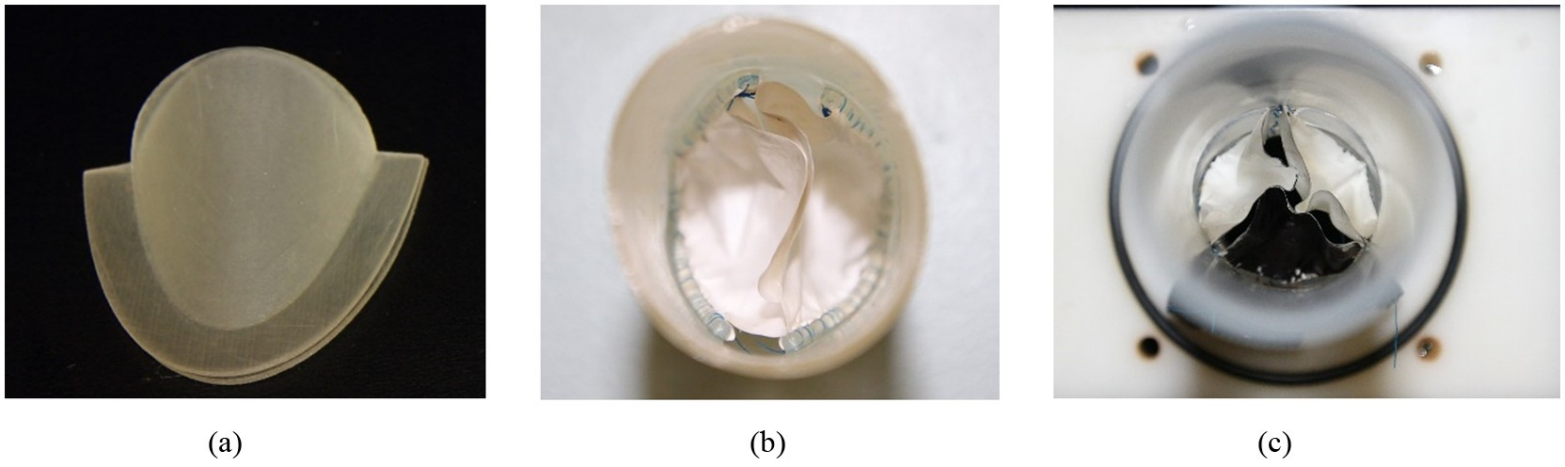
The (a) resin mold, (b) bi-leaflet valve and (c) reference tri-leaflet valve.

As described in the introduction, the preparation of the valve leaflet should be easy, steady and consistent. Thus, a set of resin molds was fabricated by using 3D printing technology (Fig 4(a)). The molds shared the same geometry of the model used in the FEM simulation. ePTFE membrane (Gore-Tex, Preclude Pericardial Membrane, W.L. Gore & Assoc., Flagstaff, AZ, USA) of 0.1 mm thickness were selected as the leaflet material. The valve leaflets were prepared by placing the ePTFE membrane in the mold and cutting along the edge. The aortic root was constructed by using a silicon polymer (VTV, MCP-HEK Tooling GmbH, Kaarst, Germany). The commissures of the leaflets were sutured to the aortic root with one running 4-0 polypropylene suture following the suture mark in the conduit (Fig 4(b)). As a reference, a fully sutured tri-leaflet valve that composed of ePTFE was also created and tested under the same *in-vitro* conditions in the experiment section (Fig 4(c)). The diameters of both valves at the base and at the commissures are 25 mm, and the overall leaflet heights of the bi-leaflet valve and tri-leaflet valve are 25 mm and 21.6 mm, respectively.

#### Experimental set-up and flow conditions

**Fig 5:**
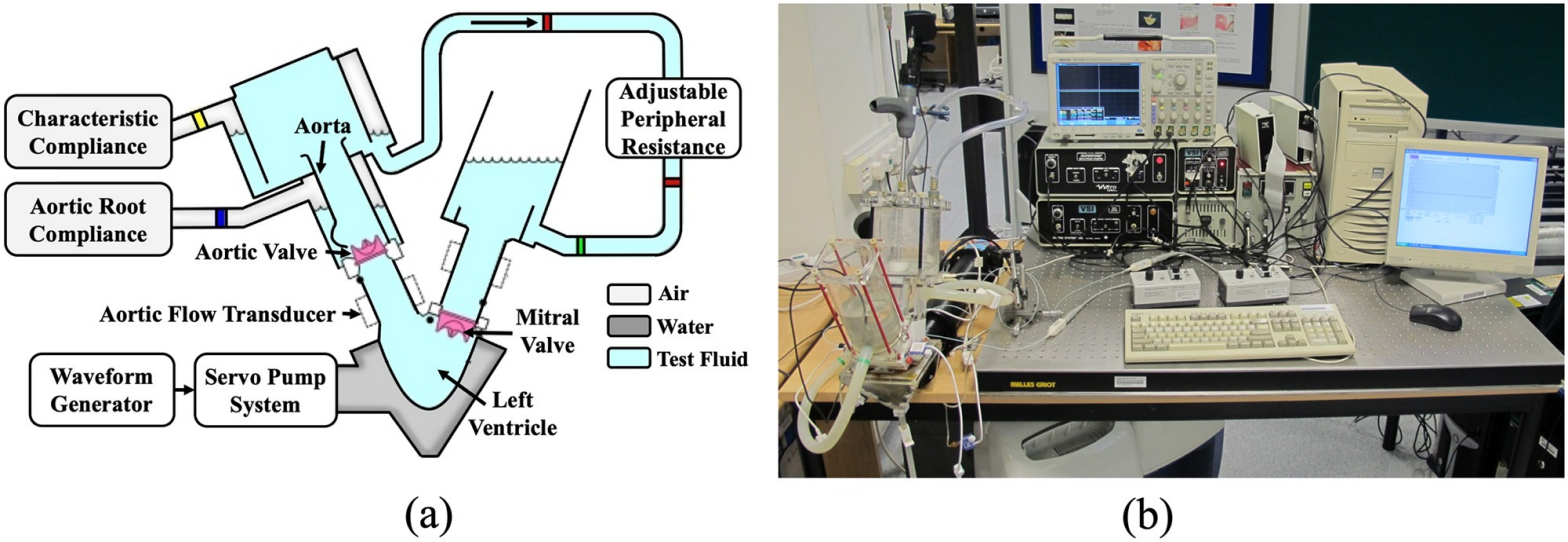
Experimental setup.

The Vivitro pulse duplicator (Vivitro Systems Inc., Victoria, BC, Canada) (Fig 5(a)) was used to generate physiological pressure and flow in the left ventricle and aorta. Two Millar MIKRO-TIP Pressure transducers (SPC 330A, Millar Instruments, Inc., Houston, TX, USA) were placed in the left ventricle and ascending aorta 10 mm above the commissural level to monitor the trans-valvular pressure gradient. The dynamic behaviors of the valve leaflet were captured by using a high-speed camera (FASTCAM-PCI R2 model 500, Photron USA, Inc., San Diego, CA, USA). The frame rate was set to 250 fps. The flow through the aortic site was measured by an electromagnetic blood flow meter (501D, Carolina Medical Electronics, East Bend, NC, USA). The experimental setup is illustrated in Fig 5(b).

The ventricular and aortic pressures were measured at the exit of the left ventricle and the exit of the aorta model. The pressures were controlled by adjusting the resistor and piston movement magnitude. The systolic and diastolic pressures in the aorta are 120 mmHg and 80 mmHg, respectively.

Flow profiles were measured by the flow probe placed at the exit of the left ventricle and were used to calculate the flow speed, cardiac output and regurgitation. All tests were conducted at a stroke volume of 75 ml (5.4 L/min) and a heart rate of 72 beats/min.

#### Working fluid

An aqueous solution of glycerol (42% by weight) was used as the working fluid to mimic blood. The dynamic viscosity and density of the working fluid were 3.52 mPa⋅s and 1038 kg/m^3^, respectively.

#### Data acquisition

TTL signals generated by the amplifier of a Vivitro system were used as trigger signals. The flow meter, pressure transducer and high-speed camera were synced by the trigger signal. Before data collection, the system was allowed to run until all readings were stable to avoid measurement errors.

## Results

### FEM simulation

#### Dynamic behaviors

**Fig 6:**
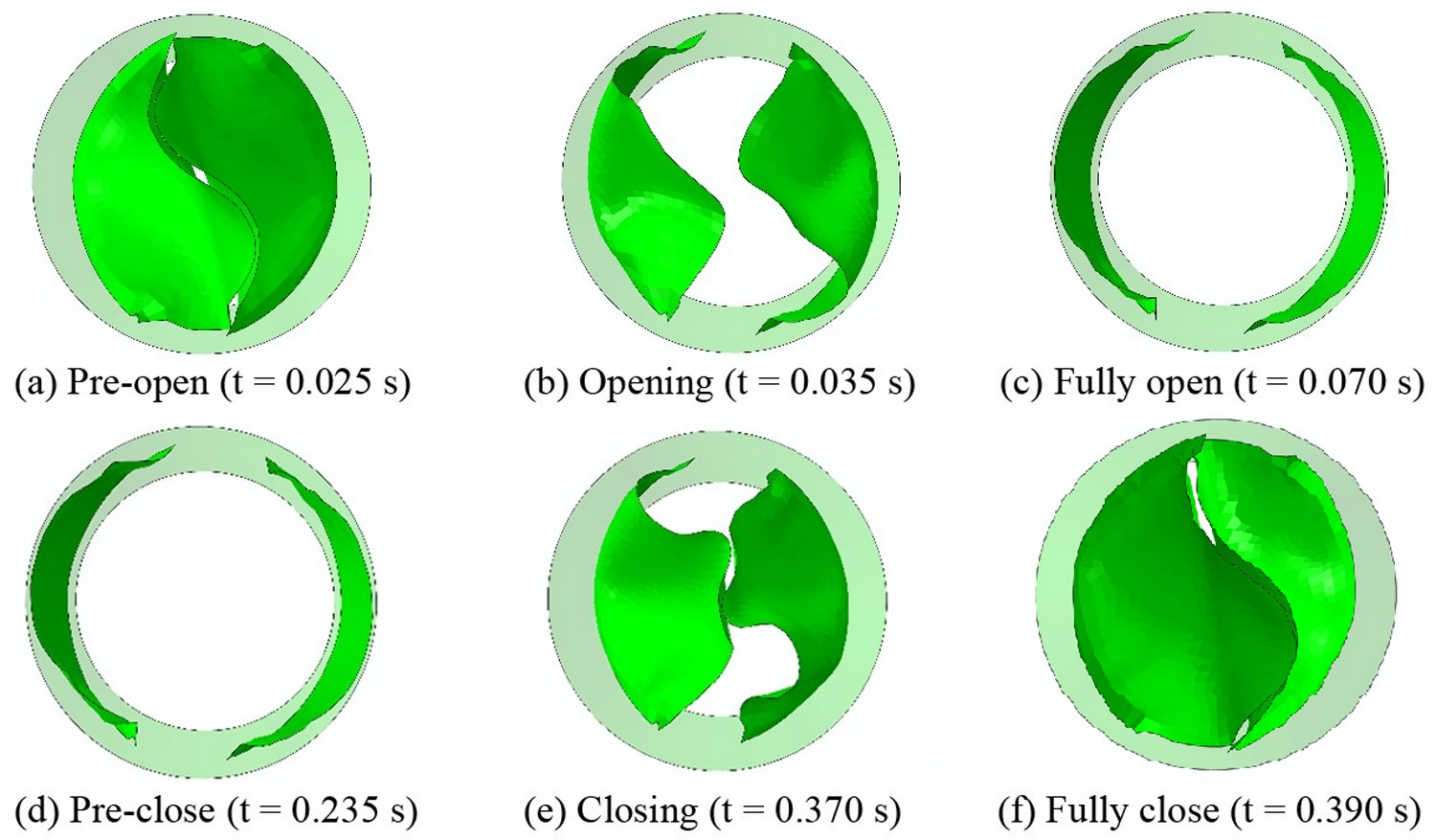
Selected frames of the dynamics deformation during a full cardiac cycle for the proposed leaflet design.

The dynamic behaviors of the leaflets were analyzed in the FEM simulation. During a full cardiac cycle, the closing phase of the bi-leaflet valve is 0.055 s, and the fully closed state is maintained for 0.41 s. The leaflets require 0.04 s to reach the fully opened position, and the fully open state lasts 0.325 s. Fig 6 shows the dynamic displacement of the leaflets in a full cardiac cycle.

#### Coaptation parameters

**Fig 7:**
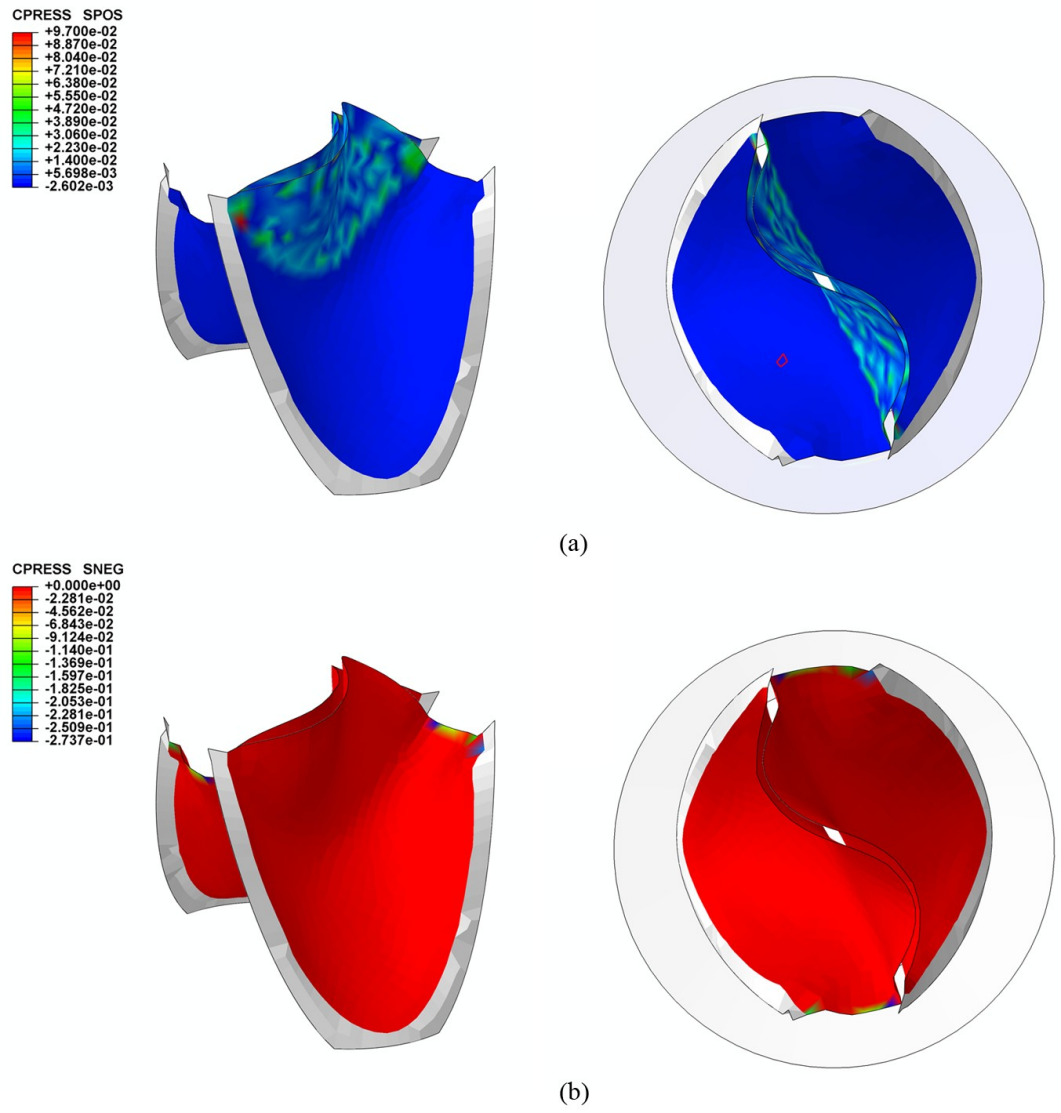
Contact pressure (a) between leaflets and (b) between leaflets and conduit.

The contact pressure normal to the leaflet was used to indicate the state of contact. A negative contact pressure indicated that the leaflet was in contact with the conduit, and a positive contact pressure implied that the leaflets were in contact with each other. Fig 7 shows the distribution of the contact pressure on the leaflets in the fully closed position. It is clear that the entire free edges of the leaflets are in the contact state at this position, and no leakage area was found.

**Fig 8:**
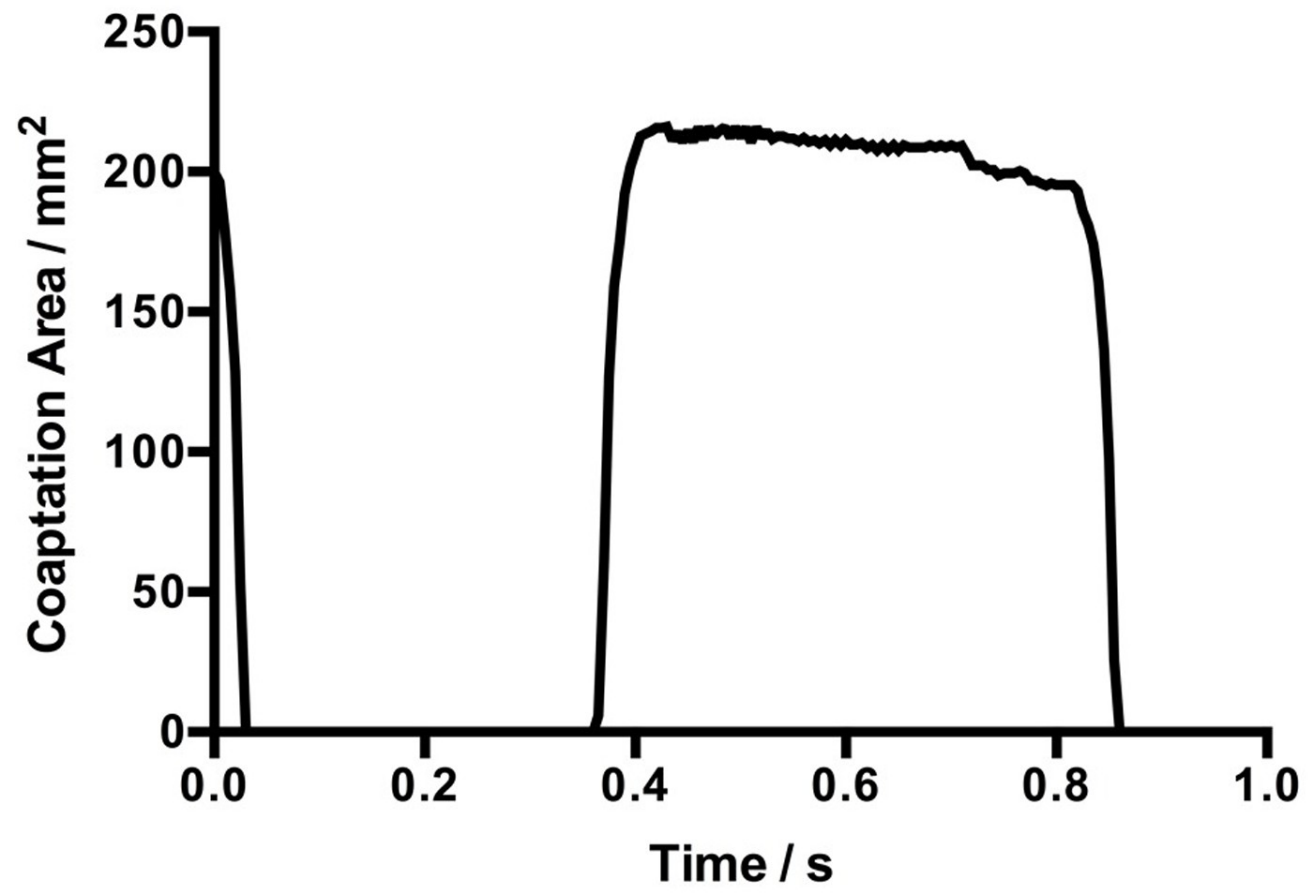
Coaptation area of a single leaflet over the cardiac cycle.

The coaptation area of a single leaflet over a cardiac cycle is plotted in Fig 8. The maximum coaptation area of the proposed design is 216.3 mm^2^.

**Fig 9:**
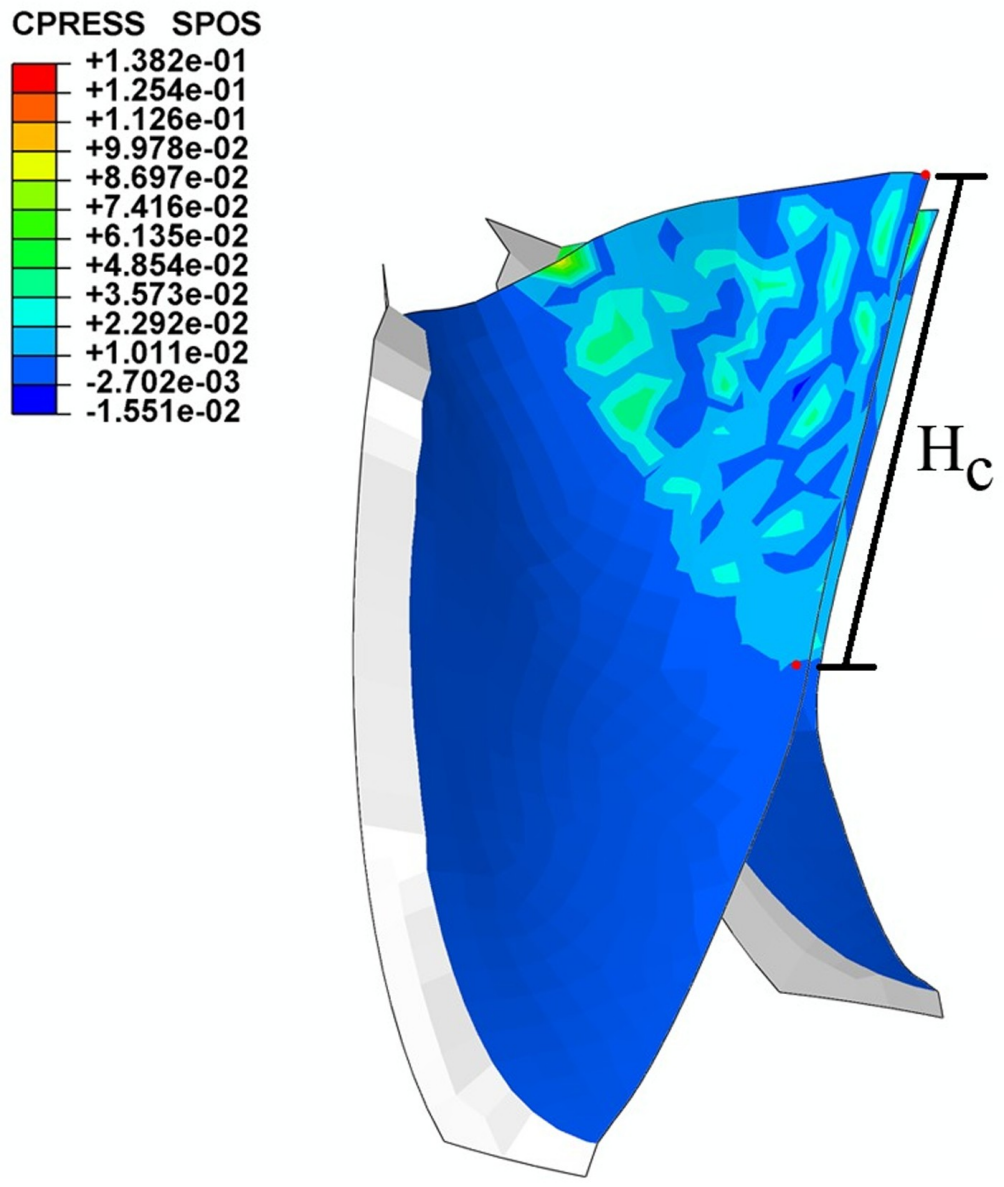
Coaptation height of the bi-leaflet valve model.

The maximum coaptation height of the proposed valve leaflet is 13.37 mm (Fig 9).

#### Leaflet stress distribution

**Fig 10:**
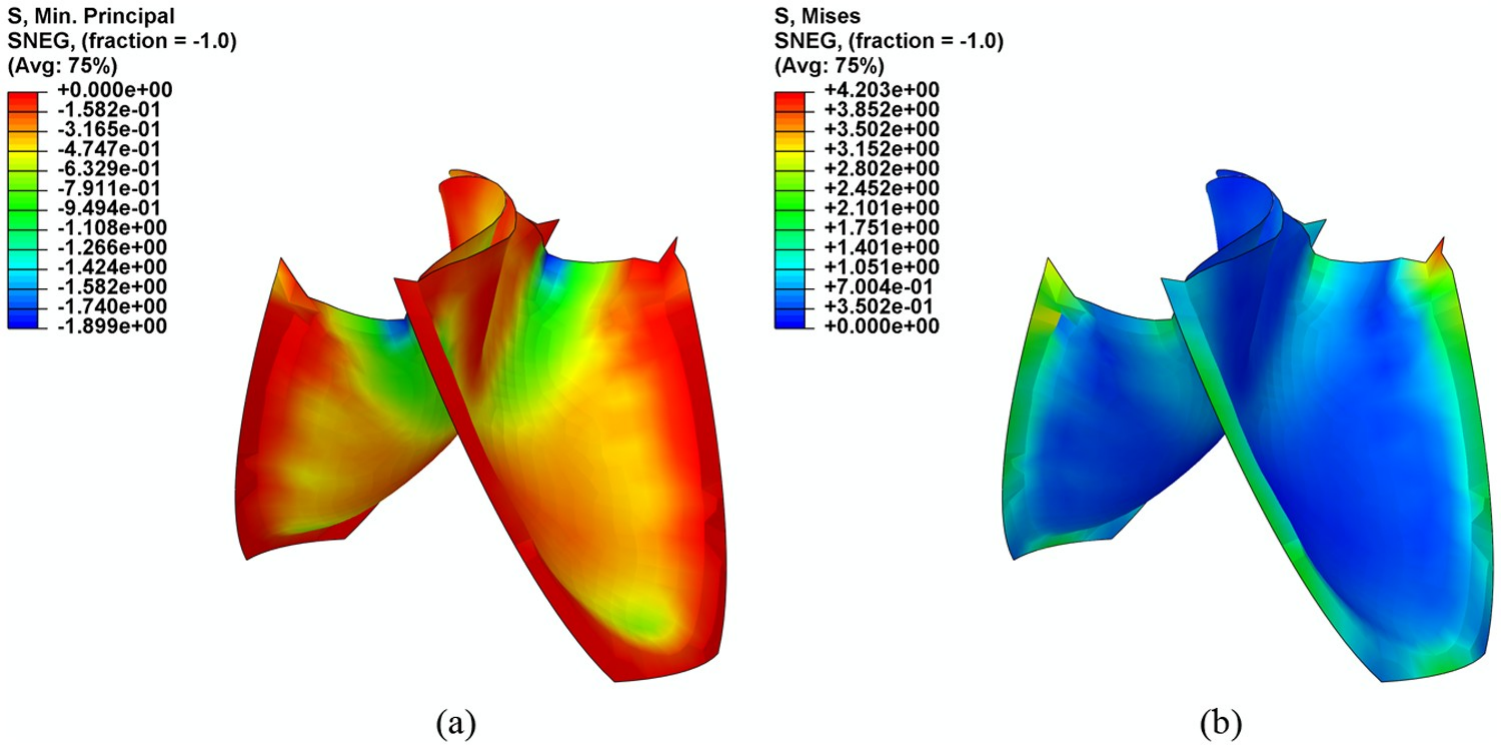
Distributions of (a) compressive stress and (b) Von Mises stress on leaflets.

Fig 10(a) and 10(b) showed the distributions of compressive stress and Von Mises stress for the proposed bi-leaflet design at the fully closed position during the maximum stress magnitudes observed. The greatest compressive stress occurs at the bending site of the leaflets, which is 1.90 MPa. High Von Mises stress exists along the commissures, and the maximum Von Mises stress of 4.29 MPa appears at the corner of the commissures.

### *In-vitro* performances

**Fig 11:**
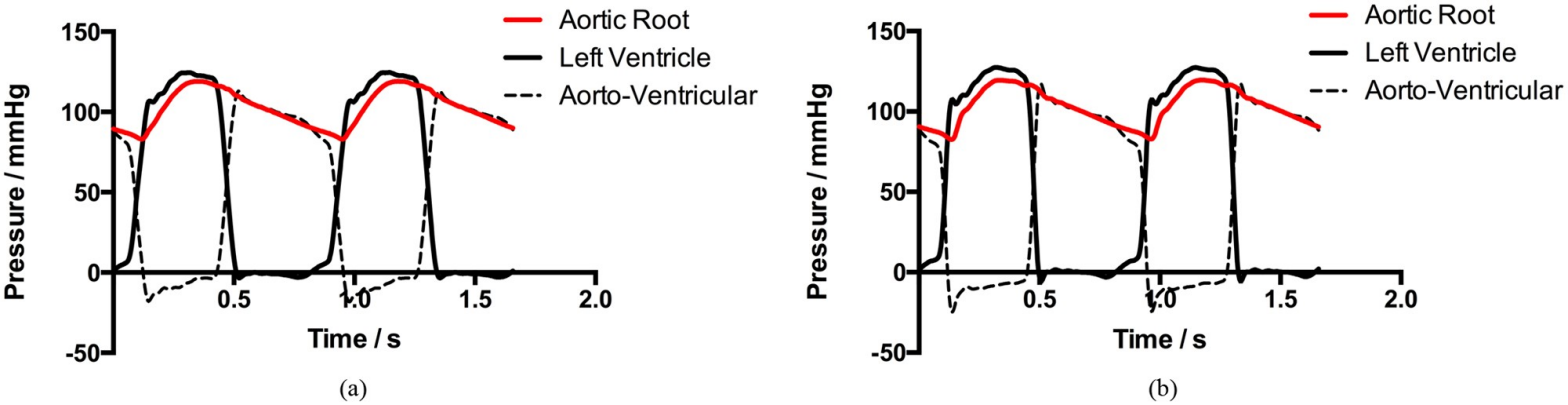
Time-varying pressure loadings of (a) bi-leaflet valve and (b) reference tri-leaflet valve measured during the *in-vitro* experiment.

The *in-vitro* performance of the proposed bi-leaflet valve and reference tri-leaflet valve were assessed under the same experimental conditions. Fig 11 shows the left ventricular pressures, aortic pressures and trans-valvular pressures of the two valves.

#### Dynamic behaviors

**Fig 12:**
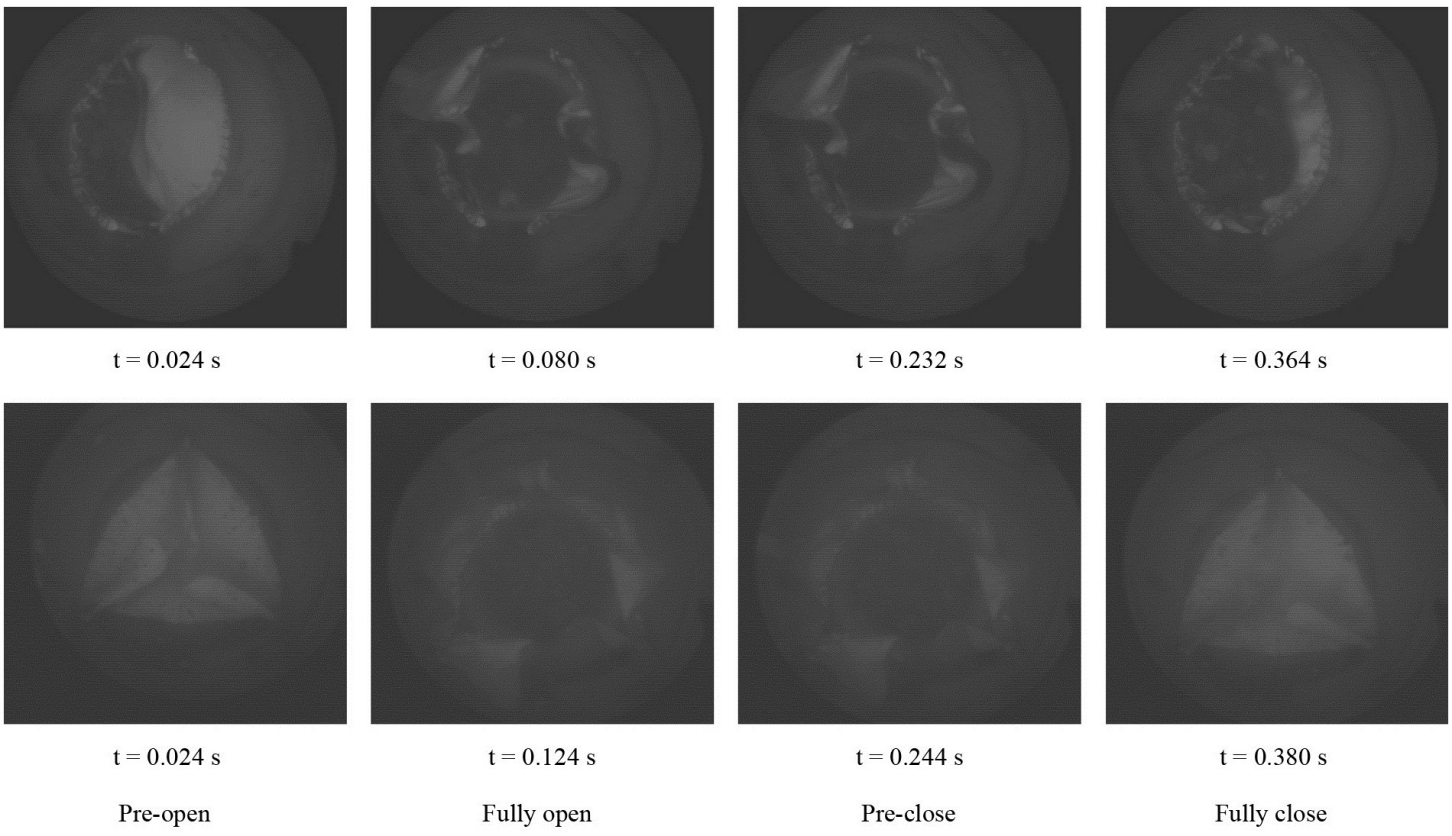
Dynamic deformation of the proposed bi-leaflet valve (top) and reference valve (bottom).

To analyze the structural dynamics, key frames from the film recorded by the high-speed camera were extracted (Fig 12).

The starting point of the recording was defined as t = 0. The opening of the leaflets begins at t = 0.024 s for both valves tested. The opening stage, fully opened stage, and closing stage of the bi-leaflet valve and the reference valve are 0.056±0.00 s and 0.1±0.00 s, 0.152±0.00 s and 0.12±0.00 s, 0.132±0.00 s and 0.136±0.00 s, respectively. The leaflets of the proposed bi-leaflet and the reference valve closed fully at 0.364 s and 0.38 s, respectively (Table 1).

**Table 1:** Comparison of structural dynamic behaviors between bi-leaflet and tri-leaflet valve. (Mean ± SD).

	Proposed bi-leaflet valve	Reference tri-leaflet valve	P
*Opening* (*s*)	0.056 ± 0.00	0.10 ± 0.00	<0.001
*FullyOpened* (*s*)	0.15 ± 0.00	0.12 ± 0.00	<0.001
*Closing* (*s*)	0.13 ± 0.00	0.14 ± 0.00	<0.01

#### Hemodynamic performance

**Fig 13:**
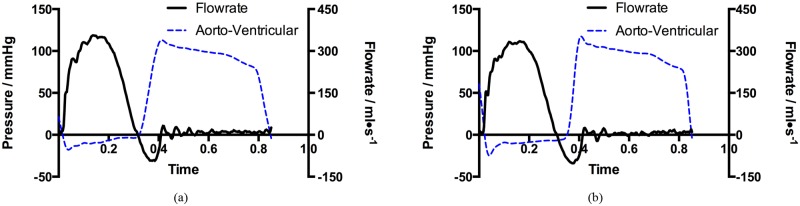
Trans-valvular prssures and aortic flow rates of the (a) bi-leaflet valve and (b) reference tri-leaflet valve over one cardiac cycle.

The mean trans-valvular pressure of the proposed bi-leaflet valve during the systolic phase is 8.74 mmHg, which is approximately 7.6% lower than that of the reference valve. The trans-valvular pressure and the aortic flow of the valves are shown in Fig 13.

The regurgitant volume (V_*R*_) and leakage volume (V_*L*_) were 5.93 ml and 1.55 ml per cycle for the proposed bi-leaflet valve and 7.09 ml and 2.81 ml for the reference valve, respectively. Thus, the regurgitant fraction (RF) can be calculated by using Eq (1):

$$\begin{array}{c} \text{RF}=\frac{V_{R}+V_{L}}{V_{F}}\times 100\%, \end{array}$$ (1)

where V_*R*_ is the regurgitant volume, V_*L*_ is the leakage volume and V_*F*_ is the forward volume.

The equation from ISO: 5840:2005 [33] was applied to evaluate the maximum EOA Eq (2):

$$\begin{array}{c} \text{EOA}=\frac{Q_{RMS}}{51.6\sqrt{\Delta P/\rho }}, \end{array}$$ (2)

where EOA is the effective orifice area of the valve (cm^2^), Δ*P* is the mean systolic trans-valvular pressure gradient (TPG) in mmHg, *ρ* is the working fluid density (g/cm^3^), and Q_*RMS*_ is the root mean square volumetric flow rate (ml/s) (Eq (3)).

$$\begin{array}{c} Q_{\text{RMS}}=\sqrt{\frac{\int_{t_{1}}^{t_{2}}Q{\left(t\right)}^{2}dt}{t_{2}-t_{1}}}, \end{array}$$ (3)

Performance index (PI), which represent the normalized resistance of the valve [34], was evaluated by using Eq (4):

$$\begin{array}{c} \text{PI}=\frac{EOA}{A_{sew}}, \end{array}$$ (4)

Derived from the Bernoulli equation, the energy loss of the left ventricle that is associated with the valve prosthesis was calculated by integrating the aorto-ventricular pressure times the flow rate with respect to time [31, 32, 35] (Eq (5)):

$$\begin{array}{c} E_{L}=0.1333\int_{t_{0}}^{t_{1}}\Delta p(t)Q\left(t\right)dt, \end{array}$$ (5)

where E_*L*_ is the energy loss (mJ), t_0_ to t_1_ is the range of a cardiac cycle, Δ*p* is the aorto-ventricular pressure difference (mmHg) and Q(t) (ml/s) is the volume flow. The calculated parameters are listed in Table 2.

**Table 2:** *In-vitro* results of the hemodynamics parameters. (Mean ± SD).

	Proposed bi-leaflet valve	Reference tri-leaflet valve	P
*V*_*R*_ (*ml*/*beat*)	5.93 ± 0.20	7.09 ± 0.15	<0.001
*V*_*L*_ (*ml*/*beat*)	1.55 ± 0.04	2.81 ± 0.03	<0.001
*RF* (%)	10.26 ± 0.00	14.37 ± 0.00	<0.001
*TPG* (*mmHg*)	8.74 ± 0.07	9.89 ± 0.07	<0.001
*E*_*L*_ (*mJ*)	98.99 ± 7.94	129.03 ± 6.34	<0.001
*EOA* (*cm*^2^)	3.14 ± 0.02	2.86 ± 0.01	<0.001
*PI*	0.64 ± 0.00	0.58 ± 0.00	<0.001

## Discussion

The goal of the current study was to develop a reliable bi-leaflet valve for patients who need AVR. A novel design of the bi-leaflet valve was proposed in the current study. The dynamic and hemodynamic performances of the newly designed valve were assessed in FEM simulations and *in-vitro* experiments. The results were compared with the reference valve that was tested under the same conditions.

### Verification of the bi-leaflet valve design

Before further discussion, it is necessary to verify that the performance of the current design complies with the technical standard. As the well-accepted industry standard, ISO 5480:2055 provides a full set of criteria for evaluating a valve design [33]. The criteria that related to the current study are listed in Table 3.

**Table 3:** Minimum performance requirements for aortic valve prostheses.

Valve size (TAD, mm)	19	21	23	25	27	29	31
*EOA* (*cm*^2^)	≥ 0.7	≥ 0.85	≥ 1.00	≥ 1.20	≥ 1.40	≥ 1.60	≥ 1.80
*RF* (%)	≤ 10	≤ 10	≤ 10	≤ 15	≤ 15	≤ 20	≤ 20

The EOA and RF values of the valves tested in this study all satisfy the criteria of the standard, which provided the basis for further discussion.

### Validation of the FEM simulation

**Fig 14:**
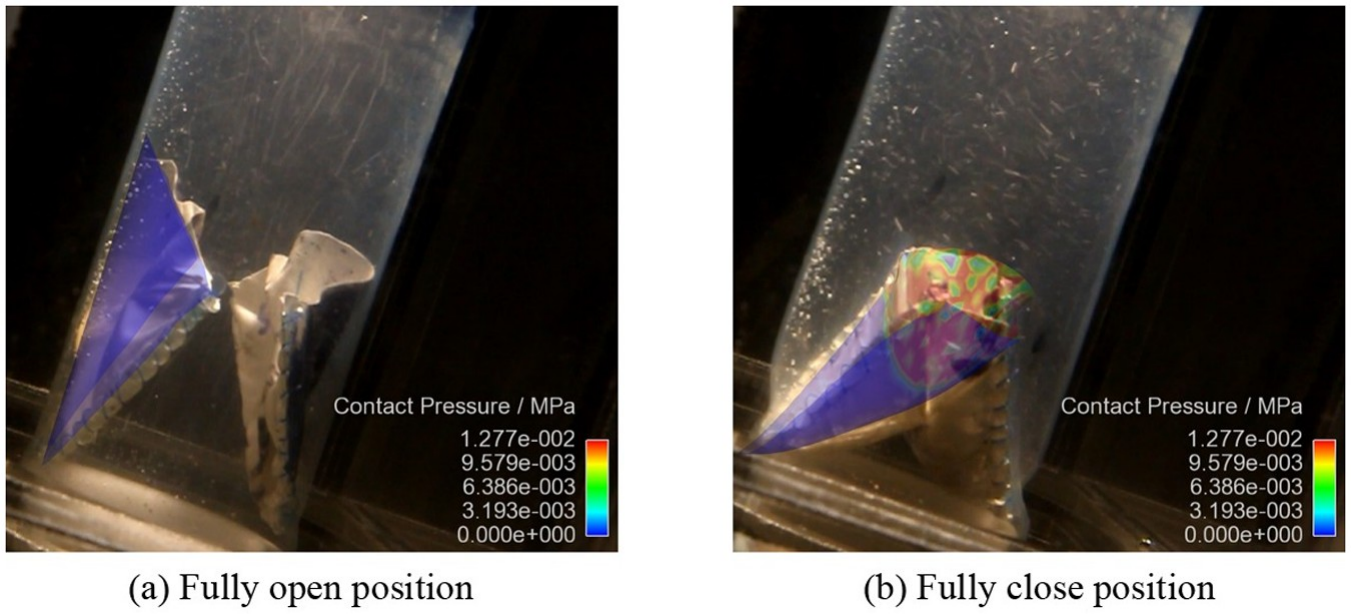
Morphological comparison of the FEM model and the *in-vitro* model in fully opened and fully closed positions.

The dynamic behavior of the leaflets is a key characteristic for assessing the performance of an aortic valve design [29]. Thus, the dynamic behavior of the valve was selected as the validation criterion. Dynamic deformation of the FEM model and the *in-vitro* model was compared in this section. Fig 14 shows a morphological comparison between the two models.

**Fig 15:**
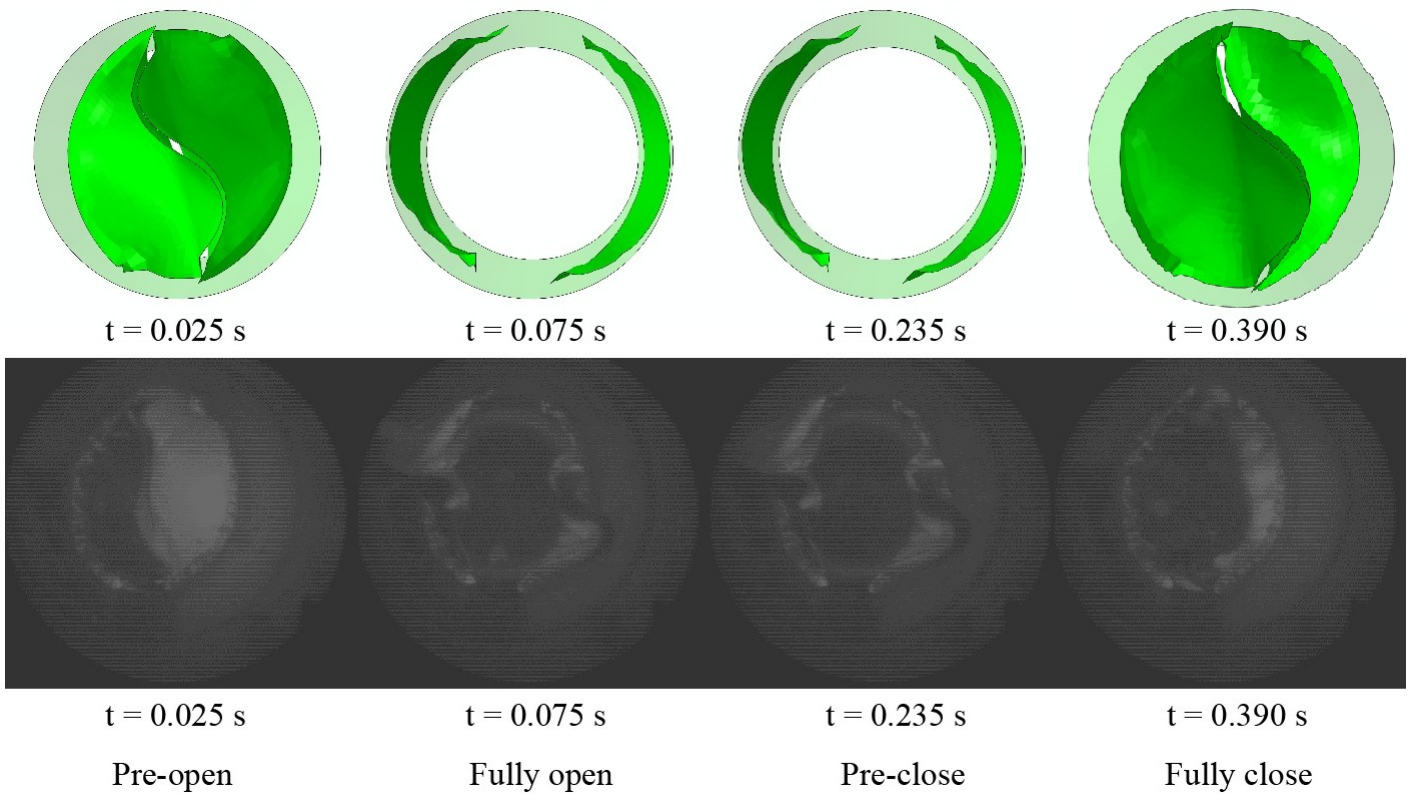
Dynamic process of the FEM and *in-vitro* model.

Fig 15 shows the dynamic processes of the models. After fully closing, S-shaped free edges were observed in the FEM model and the *in-vitro* model.

Table 4 lists the specific times required for different phases of the FEM and *in-vitro* models.

**Table 4:** Time required for different phases of the FEM and *in-vitro* models.

Phases	Cost of time / s		Proportion of a cardiac cycle / %		Difference / %
	in-vitro *model*	FEM model	in-vitro *model*	FEM model	
Opening	0.056 ± 0.00	0.050	6.747	6.024	12
Fully open	0.152 ± 0.00	0.160	18.313	19.277	-5
Closing	0.132 ± 0.00	0.155	15.904	18.675	-14

During the *in-vitro* experiment and the FEM simulation, the same boundary conditions were used to ensure a proper comparison. The morphological characters and the dynamic behaviors of the *in-vitro* and FEM models matched well. Thus, the proposed FEM simulation has been validated to some extent.

### Coaptation and stress

The coaptation height, coaptation area and stress distribution obtained from the FEM simulation were compared with the corresponding parameters reported by our group in the FEM investigation of a fully sutured tri-leaflet valve [28, 29]. Table 5 lists these data.

**Table 5:** Coaptation and stress parameters.

	Current bi-leaflet valve	Fully sutured tri-leaflet valve	Difference / %
***H***_***c***_ **(mm)**	13.37	4.50	197.11
***A*_*c*_ (%)**	37.32	51.70	-27.81
**Max Von Mises stress (MPa)**	4.20	3.92	7.14
**Max compressive stress (MPa)**	1.90	2.13	-10.79

*H*_*c*_, max coaptation height; *A*_*c*_, percentage of the maximum coaptation area to leaflet surface area;

As a critical parameter for assessing the competence of the valve, the maximum coaptation height of the bi-leaflet valve is nearly three times greater than that of the tri-leaflet valve, which suggests the former may be more competent. However, the A_*c*_ of the bi-leaflet model is 27.81% smaller than that of the tri-leaflet model, which could be due to the different contact patterns of the designs. In the bi-leaflet design, most of the leaflet is in contact with another leaflet, and the remaining part of the leaflet is in contact with the conduit. In the tri-leaflet valve, the entire leaflet is in contact with two other leaflets, thus increasing the contact area. The impact of the reduced A_*c*_ is discussed further in a later section.

Mechanical stress has long been related to calcification and structural failure of aortic valve prostheses [36, 37]. Thubrikar et al. reported in an *in vivo* study [37] that, high compressive stress is closely correlated with the calcification of aortic valve prostheses leaflets. In addition to the compressive stress, the tensile stress and shear stress that act on leaflets may lead to the failure of the valve structure [36]. The multiple stress components can be represented by the Von Mises stress. Our current results show that the highest compressive stress occurs at the site of the leaflet that is most bent. The same pattern was also observed in the FEM model of the tri-leaflet valve. However, the maximum compressive stress of the bi-leaflet valve is 10.8% smaller than that of the tri-leaflet valve. The highest Von Mises stress value in the bi-leaflet valve is slightly higher (by 7.1%) than that in the tri-leaflet valve. The maximum Von Mises stress occurs at the commissures ends in both valves.

As mentioned in the validation section, S-shaped free edges were observed when the bi-leaflet valve was fully closed. This observation ensured good coaptation of the leaflets and provided more safety for the closure [29].

### Dynamics and hemodynamic performance

As the FEM simulation only considered structural behaviors, evaluation of the dynamics and hemodynamic performance of the bi-leaflet design was performed by comparing the *in-vitro* results. The bi-leaflet valve and reference tri-leaflet valve were compared.

The results show that while both valves are capable of normal function, the dynamic performance of the proposed bi-leaflet valve is superior to that of the reference tri-leaflet valve. The bi-leaflet design required for less time than the tri-leaflet valve in the opening and closing phase. The opening of the bi-leaflet valve is 44% faster than the reference valve. During the close phase, the time cost of the bi-leaflet design is 2.9% less than the reference valve. This observation suggested that the leaflet mobility of the proposed bi-leaflet design is better than the reference valve. The fully opened state of the proposed valve lasts 26.7% longer than that of the reference valve, corresponding to 0.152 s and 0.12 s, respectively.

In addition, the novel designed bi-leaflet valve also exhibited favorable hemodynamic performance in all aspects studied compared with the reference valve. The trans-valvular pressure measurements indicated that the pressure drop of the bi-leaflet valve is 11.63% lower than that of the reference tri-leaflet valve, which in turn implies a lower flow impedance. The RF, regurgitant volume and leakage volume of the bi-leaflet valve are 4.11%, 16.36% and 44.84% lower than those of the reference valve, respectively. Based on the directly measured parameters, the calculated EOA and energy loss of the bi-leaflet valve are 9.7% larger and 23.28% smaller than those of the reference tri-leaflet valve, respectively. The larger EOA of the proposed valve design lowers the risk of post-operative trans-valvular pressure, thus reducing the resistance to forward flow and energy loss. In addition to the results of the structural dynamic investigation, the simpler geometrically structure of the bi-leaflet valve guarantees faster coaptation, thus leads to a smaller regurgitant volume than the reference valve.

To further validate the function of the design proposed, a more extensive comparison of the hemodynamic performance was performed between the bi-leaflet valve proposed in the current study and aortic valve prostheses reported in the literature (Table 6).

**Table 6:** Comparison of hemodynamic performance between proposed bi-leaflet valve and reported aortic valve prosthesis.

Valve Type	CO	HR	Size	TPG	*EOA*	*V_L_*	*V_L_*	RF	*E_L_*	PI
Polymeric bi-leaflet	5.4	72	25	8.74 ± 0.07	3.14 ± 0.02	5.93 ± 0.20	1.55 ± 0.04	10.26 ± 0.00	98.99 ± 7.94	0.64 ± 0.00
Polymeric tri-leaflet [31]	5	-	22	3.2	3.34	1.2	6.5	-	-	-
Polymeric tri-leaflet [32]	5.6	-	21	20.91	1.47	-	-	2.43	-	-
Tissue tri-leaflet [32]	5.6	-	21	16.57	1.95	-	-	7.08	-	-
St. Jude [38]	-	-	25		1.67±0.09	-	-	-	-	-
St. Jude [39]	5.4	-	25	11	3.23	9.7	-	-	-	-
Bjork-Shiley Monostrut [39]	5.4	-	25	12	2.37	7.3	-	-	-	-
Carpentier-Edwards [39]	5.4	-	25	-	1.52	1.2	-	-	-	-
Bjork-Shiley [40]	-	-	25	4.9±3.9	3.51±1.66	-	-	-	-	0.72±0.34
Carpentier-Edwards [40]	-	-	25	9.6±7.1	2.53±1.39	-	-	-	-	0.52±0.28
Hancock [40]	-	-	25	7.9±4.1	2.23±0.74	-	-	-	-	0.46±0.15
Medtronic Hall [38]	-	-	25		1.82±0.14	-	-	-	-	-
Hancock [38]	-	-	25		1.22±0.21	-	-	-	-	-
Medtronic Open Pivot [38]	-	-	25	11.1±0.8	2.1±0.1	-	-	-	-	-
Mosaic bioprosthesis [41]	-	-	25	12.2±5.8	2.39±0.76	-	-	-	-	-
Perimount bioprosthesis [41]	-	-	25	13.7±4.4	2.07±0.35	-	-	-	-	-
Freestyle stentless bioprosthesis [41]	-	-	25	5.1±3.3	2.0±0.5	-	-	-	-	-
Perimount Magna pericardial xenograft [42]	-	-	25	7.8±1.8	2.35±0.30	-	-	-	-	-
Medtronic Mosaic bioprosthesis [42]	-	-	25	11.8±3.3	1.75±0.53	-	-	-	-	-
Perimount Magna pericardial xenograft [43]	-	-	25	8.4±2.6	2.33±0.18	-	-	-	-	-
Perimount Standard pericardial xenograf [43]	-	-	25	10.7±6.6	1.89±0.59	-	-	-	-	-
St. Jude Medical Regent [44]	-	-	25	5.8±3.4	2.5±0.9	-	-	-	-	-
Trifecta aortic bioprosthesis [45]	-	-	25	11±5		-	-	-	-	-
Trifecta aortic bioprosthesis [46]	-	-	25	4.8	2.1	-	-	-	-	-
St. Jude Toronto porcine [46]	-	-	25	-	1.9	-	-	-	-	-
Hyaluronan-Polyethylene flexible valve [47]	5	-	25		2.34±0.5	4.6±0.4	-	-	-	-
TTK Chitra tilting disc vale [48]	-	-	25	7.9±4.5	1.38±0.16	-	-	-	-	-
Terifecta aortic bioprosthesis [49]	-	-	25	6.9±2.3	2.3±0.4	-	-	-	-	-
JenaValve [50]	-	-	25	10.3±4.8		-	-	-	-	-
Tri-leaflet pericardium [51]	-	-	26	9.4±3.2	2.3±0.6	-	-	-	-	-
Trifecta aortic bioprosthesis [52]	-	-	25	7.8±3.3	-	-	-	-	-	-
Trifecta aortic bioprosthesis [53]	-	-	25	7.6	2.27	-	-	-	-	-
Medtronic Hall tilting disk [54]	5	-	25	-	3.07	4.7	4.3	10.83	-	-
St. Jude bi-leaflet [54]	5	-	25	-	3.23	5.5	5.2	12.68	-	-
Bjork-Shiley Monostrut [54]	5	-	25	-	2.62	5	4.2	9.8	-	-
Edwards pericardial [55]	-	-	25	14.0±2.6	1.8±0.2	-	-	-	-	-
Medtronic Mosaic [55]	-	-	25	15.9±2.9	1.8±0.2	-	-	-	-	-
Trifecta aortic bioprosthesis [56]	-	-	25	8.4±3.3	1.33±0.44	-	-	-	-	-
Starr-Edwards	5	70	25	-	1.62	4.3	-	-	-	0.33
Bjork-Shiley Convexo- Concave [34]	5	70	25	-	2.37	7.3	-	-	-	0.48
Bjork-Shiley Monostrut [34]	5	70	25	-	2.62	7.6	-	-	-	0.53
Medtronic Hall [34]	5	70	25	-	3.07	8.4	-	-	-	0.62
St. Jude Standard [34]	5	70	25	-	3.23	9.9	-	-	-	0.66
St. Jude Regent [34]	5	70	25	-	3.97	11.2	-	-	-	0.81
CarboMedics [34]	5	70	25	-	3.14	6.1	-	-	-	0.64
Sorin Bicarbon [34]	5	70	25	-	2.39	-	-	-	-	0.69
Carpentier-Edwards Porcine 2625 [34]	5	70	25	-	1.52	<2	-	-	-	0.31
Carpentier-Edwards Porcine 2650 [34]	5	70	25	-	2.36	<2	-	-	-	0.48
Carpentier-Edwards Pericardial 2900 [34]	5	70	25	-	3.25	<2	-	-	-	0.66
Hancock Porcine 242 [34]	5	70	25	-	1.93	<2	-	-	-	0.39
Hancock MO Porcine 250 [34]	5	70	25	-	2.16	<2	-	-	-	0.44
Hancock II Porcine 410 [34]	5	70	25	-	2.1	<2	-	-	-	0.43
Mosaic Porcine [34]	5	70	25	-	2.11	<2	-	-	-	0.43
Medtronic Freestyle Porcine [34]	5	70	25	-	3.41	<4	-	-	-	0.69
St. Jude Toronto [57]	-	-	25	9.2±3.5	1.7±0.6	-	-	-	-	-
Perimount [57]	-	-	25	6.9±4.4	2.2±0.6	-	-	-	-	-

CO, cardiac output; HR, heart rate; PI, performance

**Fig 16:**
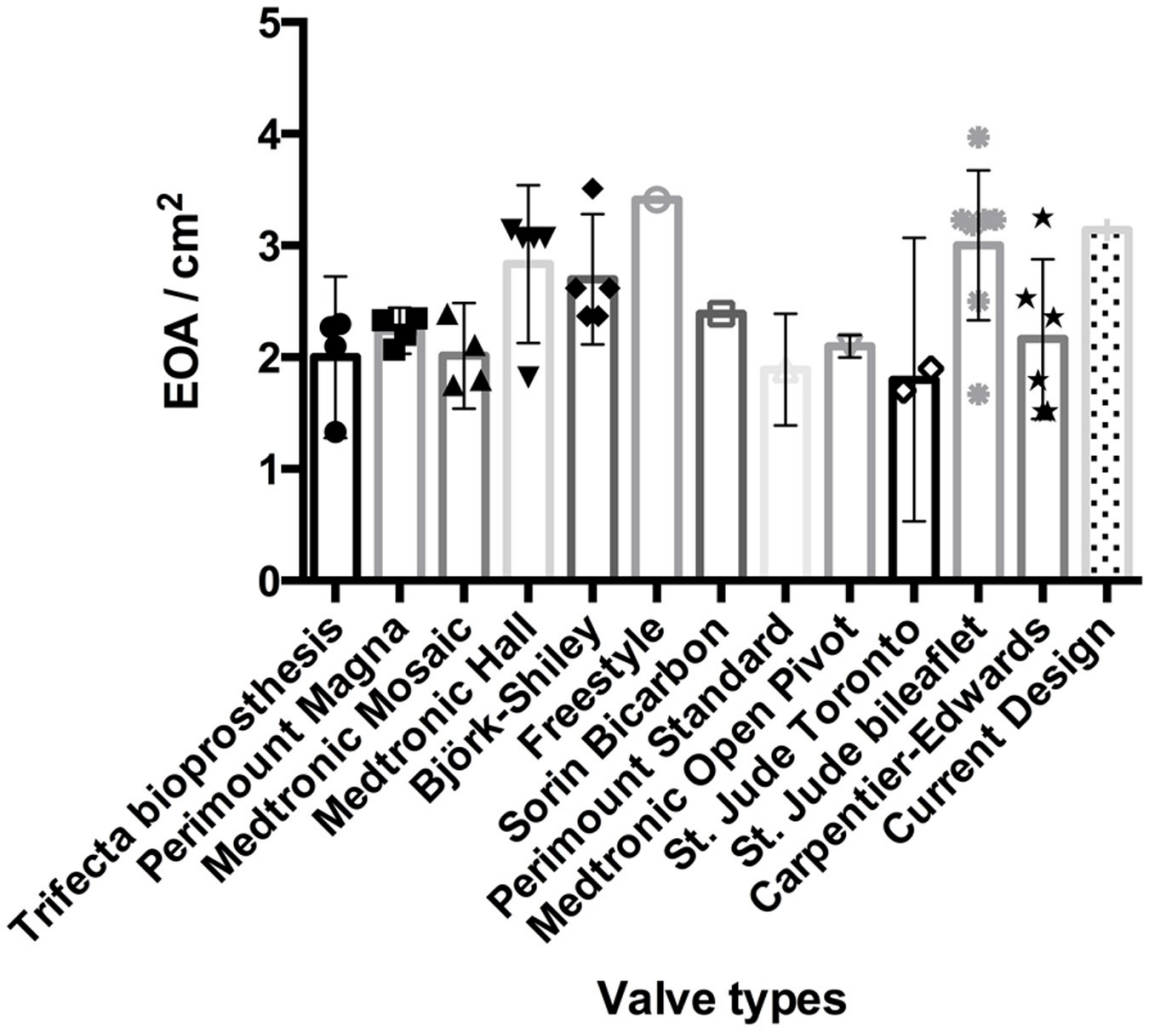
Comparison of EOA between the current design and commercially available prosthetics.

**Fig 17:**
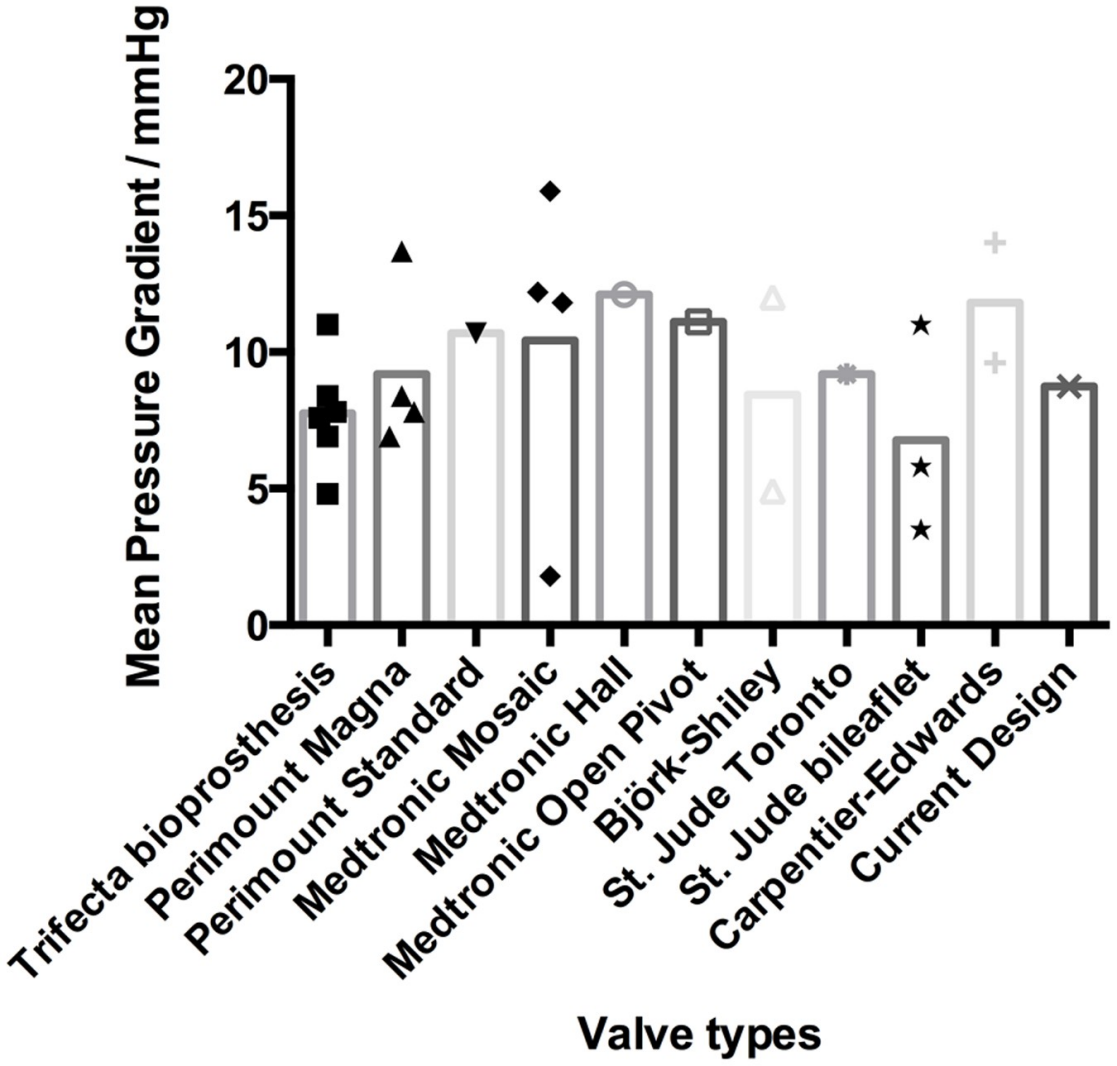
Comparison of the mean pressure gradient between the current design and commercially available prosthetics.

Two critical parameters, EOA and mean pressure gradient, were selected from the table and compared visually in Figs 16 and 17. All selected valves are sized 25 mm on the label.

As illustrated in Fig 16, the EOA of the proposed bi-leaflet valve is ranked 2^nd^ among all 13 types of valves compared. The differences in the mean pressure gradients among different valve types are not obvious, and the proposed bi-leaflet valve exhibited comparable hemodynamic performance to currently available valve designs.

**Fig 18:**
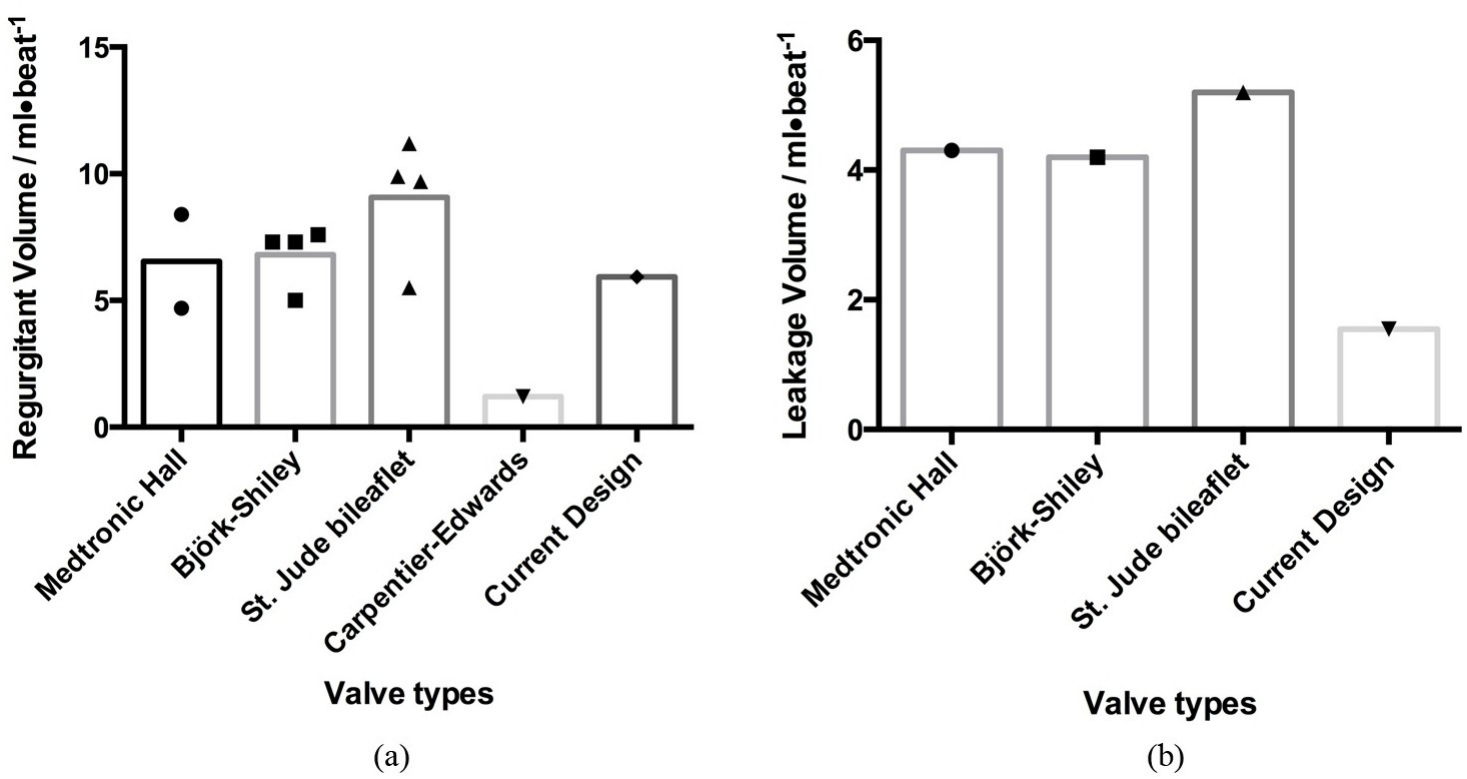
Comparison of (a) regurgitant volume and (b) leakage volume among the valves.

In addition to EOA and mean pressure gradient, the regurgitant and leakage volumes of the proposed valve were reasonable compared with available data (Fig 18).

### Impact of the bi-leaflet design on coronary flow

Coronary arteries that connect to the left and right sinuses of aortic root are responsible for supplying blood to the heart. Unlike its tri-leaflet counterparts, the impact of the unique structural characteristics of the proposed bi-leaflet prosthetic aortic valve on the coronary flow is still unclear due to the very limited data.

To clarify the impact of the proposed bi-leaflet valve on the coronary flow, CFD simulations were conducted to investigate the coronary perfusion at t = 0.4 s of Fig 13, of which is the beginning of the diastolic of the left ventricle, and the maximum coronary flow can be expected around this point under physiological conditions.

**Fig 19:**
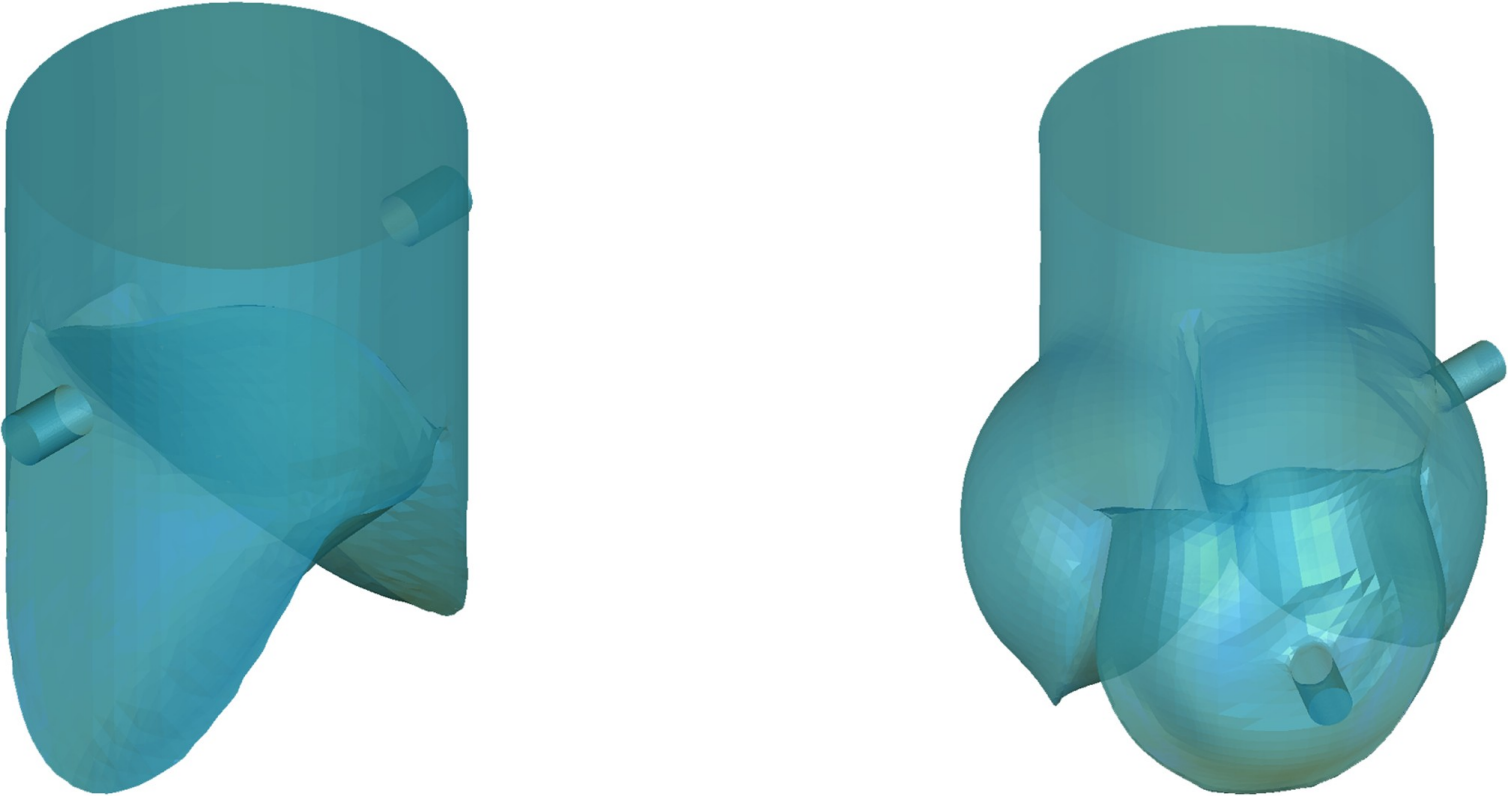
Modeling of the conduits with coronary arteries.

In the simulations, the coronary arteries were added to the conduits of the bi-leaflet model and the tri-leaflet model, respectively (Fig 19).

**Fig 20:**
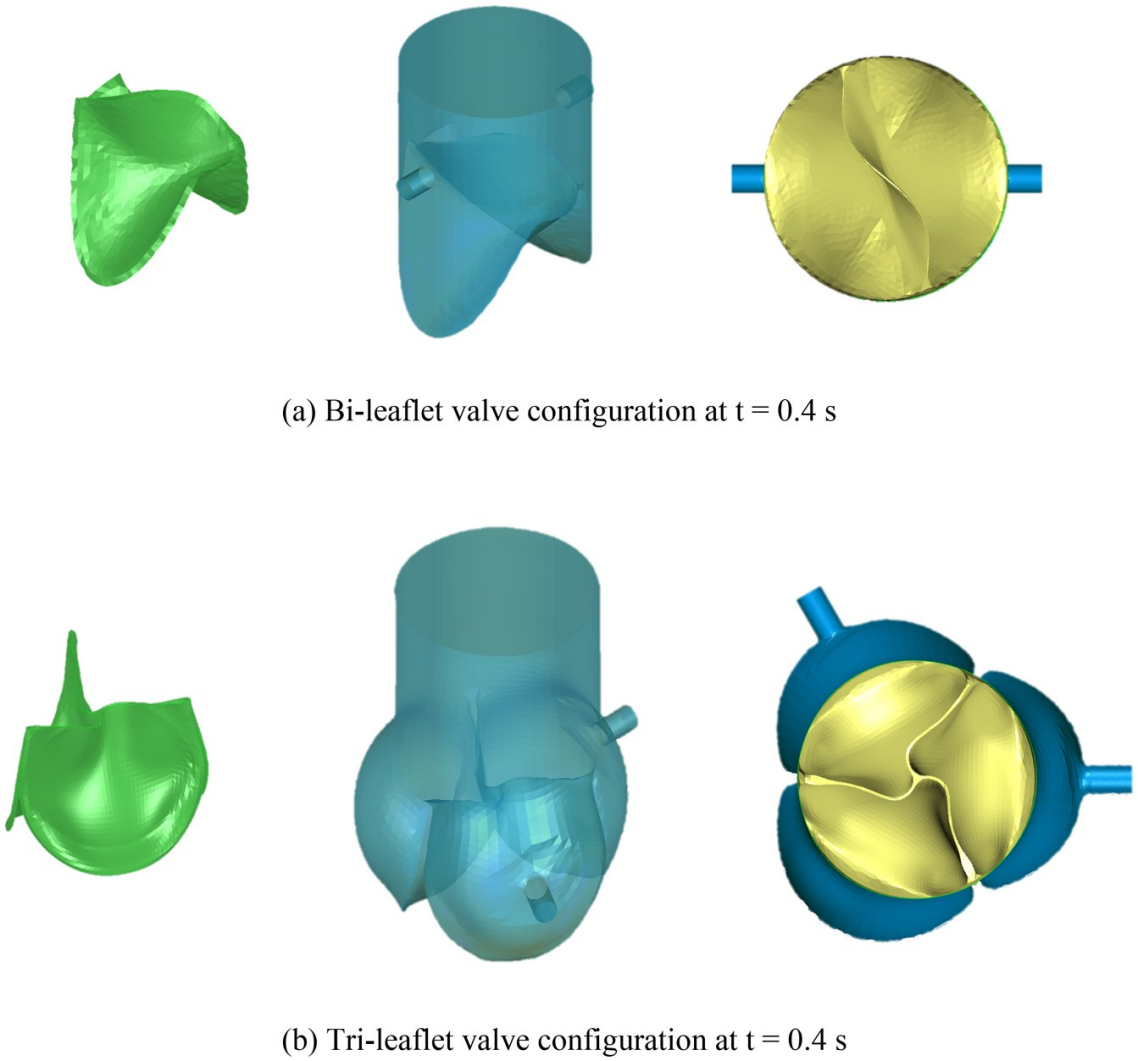
Diagram of valve configurations at t = 0.4 s.

The deformed bi-leaflet valve and tri-leaflet valve at t = 0.4 s were extracted from the FEM simulation results and incorporated into the their own conduits, respectively (Fig 20).

**Fig 21:**
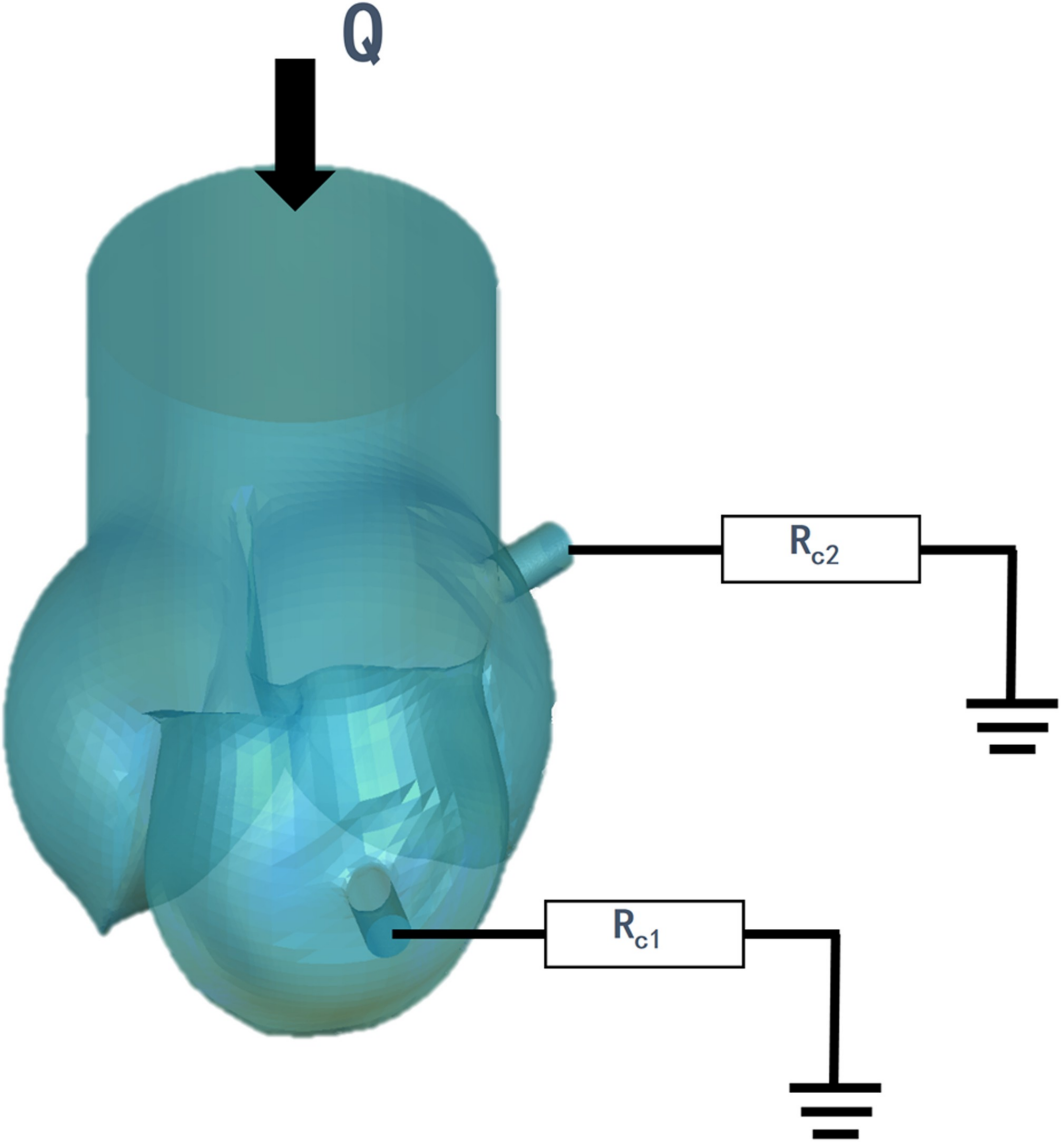
Diagram of the boundary conditions.

The in-vitro measured flow rate (-6.2 ml/s) was assigned to the distal ends aorta as inlet boundary conditions. Lump parameter resistance *Rc*_1_ and *R*_2_ were assigned to the end of left and right coronary arteries, respectively (Fig 21). The *Rc*_1_ is 34625 dyne⋅s/cm^5^ and the *Rc*_2_ is 40338 dyne⋅s/cm^5^. Due to the current CFD simulations were performed under steady-state conditions, the authors did not consider the distal vascular compliance in the lumped parameter models.

In the CFD simulation, rigid wall assumption was assumed. The fluid was model as a Newtonian fluid with a dynamic viscosity of 3.5 mPa⋅s and a density of 1040 kg/m^3^.

The flow rates at the ends of coronary arteries were monitored on both models and listed in (Table 7).

**Table 7:** Coronary flow rates of both models.

Model	Flow rate (ml/s)	
	Left coronary	Right coronary
**Bi-leaflet**	3.36	2.84
**Tri-leaflet**	3.31	2.89

Based on the CFD simulation, the differences in coronary flow between the bi-leaflet model and tri-leaflet model are small than 2%. The bi-leaflet valve shows no noticeable impact on the coronary flow compared with its tri-leaflet counterpart.

### Impact of the bi-leaflet design on the flow in aorta

The flow in ascending aorta is highly affected by the aortic valve. Abnormal flow characteristics, such as eccentric jet and stress distribution, play important roles in the development of ascending aorta dilation [58–60].

Despite the proposed bi-leaflet valve prosthesis is well-designed for AVR that open and close in a symmetrical manner, particle image velocimetry (PIV) measurements were conducted on the bi-leaflet and reference tri-leaflet prosthesis to further verify the downstream flow characteristics.

During the measurement, the PIV was triggered at the systolic peak and 60 pairs of PIV images were captured in the central of ascending aorta. The images were carefully calibrated and post-processed in the DaVis software (LaVision, Germany). The adaptive correlation method calculates velocity vectors within an initial interrogation area (IA) of 32 x 32 pixels with 50% overlapping.

**Fig 22:**
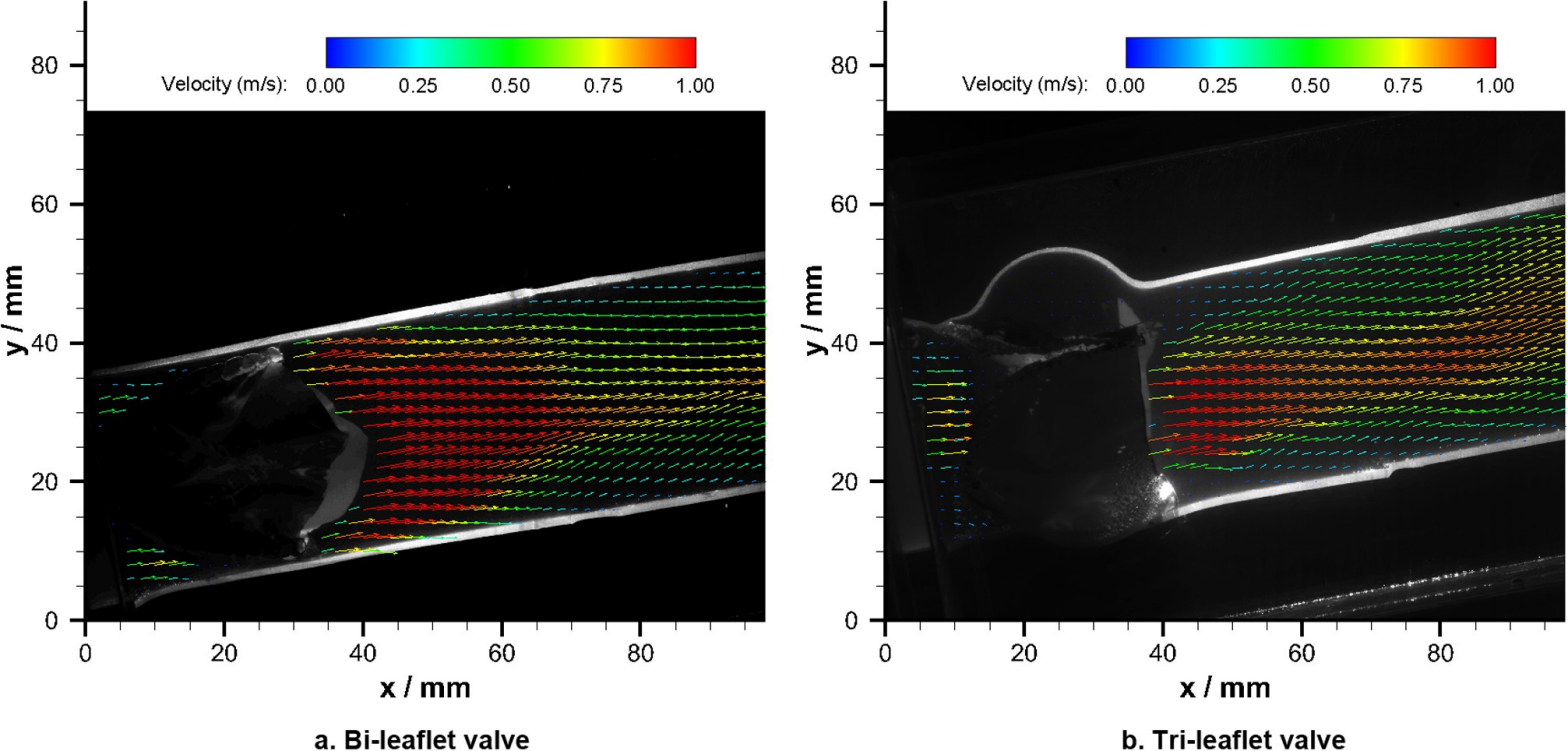
Velocity fields in the ascending aorta.

Fig 22(a) illustrated the velocity field downstream of the bi-leaflet valve at the systolic peak. Similar to its tri-leaflet counterpart(Fig 22(b)), no eccentric jet was observed.

**Fig 23:**
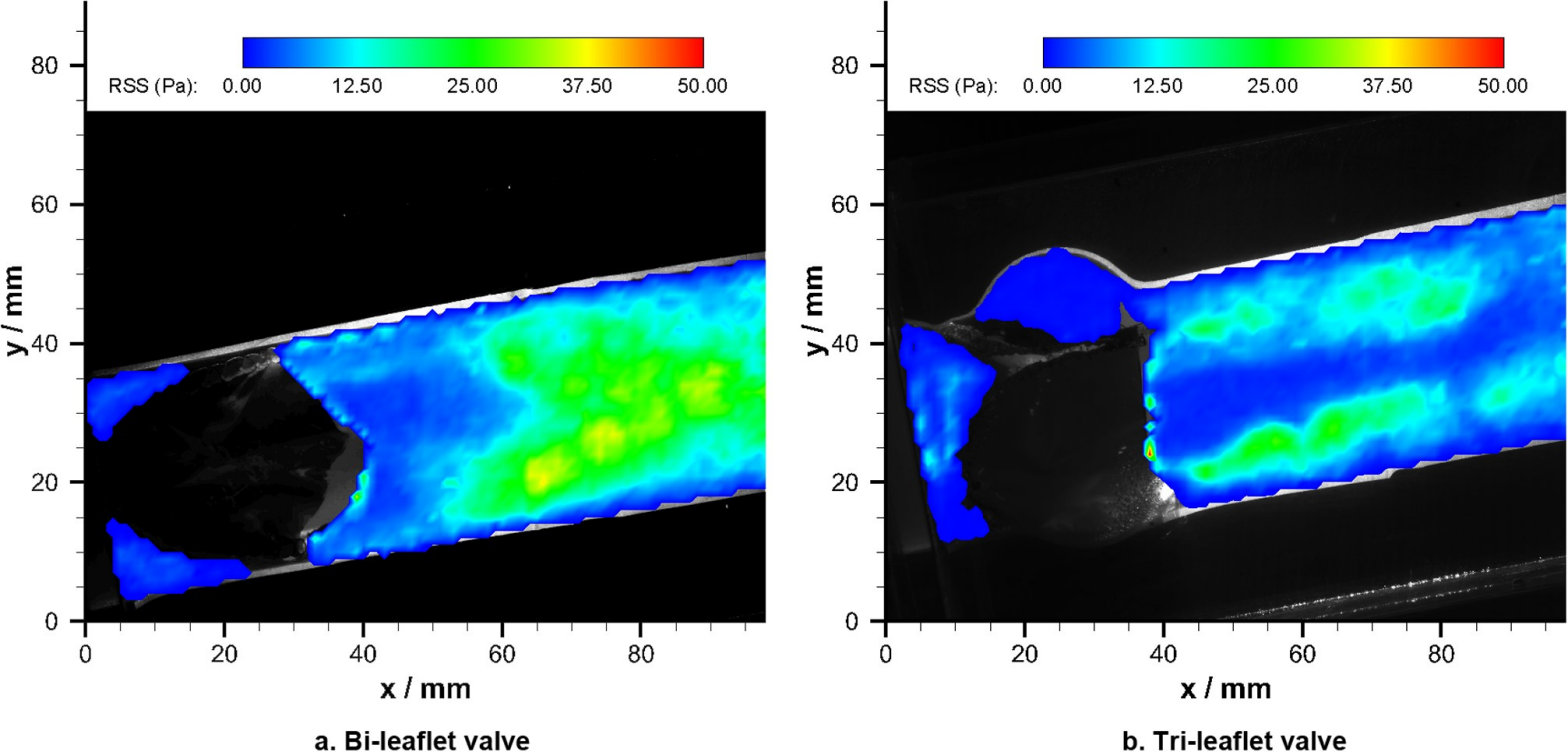
Shear stress distributions in the ascending aorta.

Based on the velocity fields, the shear stress distribution downstream the bi-leaflet valve and the reference tri-leaflet valve was calculated, respectively (Fig 23). Similar shear stress levels were observed in the ascending aortas.

### Limitations

The investigation on the feasibility of bi-leaflet ePTFE aortic valve prosthesis is still in the very early stage. Despite the bi-leaflet concept has been used as PVR prosthesis in the RVOT reconstructions successfully, some important investigations are yet to be carried out before the clinical application of the proposed prosthesis in AVR operations due to the pathological and hemodynamics environment in aorta differ from that in the pulmonary artery.

Firstly, the long-term durability of the ePTFE prosthetic valve under aortic loading is yet to be explored by high cycle fatigue tests. Additionally, systemically *in-vitro* investigations of the impact of the sinuses of Valsava (height, depth, etc.,) on the local hemodynamics and the dynamic performance of the bi-leaflet valves would be necessary for the next stage of the study. Thirdly, the impact of the bi-leaflet aortic substitute on coronary perfusion and downstream flow is largely unknown, and detailed investigations should be conducted in the next stage. Last but not least, animal trials would be required previous to any clinical applications.

## Conclusion

In conclusion, this study presents a novel ePTFE bi-leaflet valve prosthesis for AVR, and the dynamic and hemodynamic performance of the proposed bi-leaflet valve under physiological aortic loading were evaluated by using numerical and *in-vitro* experimental methods. The preliminary results showed that the bi-leaflet valve design is not only capable of serving as an aortic valve substitute under aortic physiological loadings in terms of structural dynamic behaviors, but also shows encouraging outcomes in certain critical hemodynamic parameters, including EOA, TPG, and RF when comparing with its commercialized counterparts. These novel findings could have implications for the further studies on the use of the ePTFE bi-leaflet valve in the pediatric patients who need AVR.

## Supporting information

S1 Video The motion of the bi-leaflet valve cusps under aortic flow conditions. (MOV)

S2 Video The dynamic behavior of the bi-leaflet valve captured by high speed camera. (MOV)
